# Beyond mediation: an evolutionary benchmark for emotionally and normatively competent AI

**DOI:** 10.3389/frai.2026.1719225

**Published:** 2026-04-23

**Authors:** Kohei Oshio

**Affiliations:** Graduate School of Information and Communication, Meiji University, Tokyo, Japan

**Keywords:** agent-based simulation, mediation support, negotiation dynamics, AI mediator policies, emotional regulation, trust repair, interaction-style asymmetry, evolutionary selection

## Abstract

This study presents an agent-based simulation framework for assessing the adaptive and emotional abilities required of AI systems acting as mediators in social and legal environments, where being right does not necessarily mean achieving fairness and stability. Unlike benchmarks based on logical inference or computational efficiency, the framework measures three key traits of effective dispute resolution: emotional regulation, willingness to compromise, and post-conflict trust repair. We defined five canonical negotiation tasks that vary in psychological reactivity, escalation, fatigue in compromise, and interaction-style asymmetry (S1, H1, H2, H3, C1), comparing five mediator policies under identical stochastic conditions using independent Monte Carlo episodes per condition with pre-specified endpoints: agreement probability, conditional time to agreement, inequality of outcomes, robustness across tasks, and a composite score with pre-specified weights that makes efficiency–equity–stability trade-offs explicit. An evolutionary analysis examined long-run policy selection. The results revealed stable trade-offs across scenarios: strong control shortens negotiations yet systematically worsens inequality and does not reliably increase agreement, whereas transparent, weak interventions more consistently balance efficiency and fairness across heterogeneous conditions; slight, reversible framing improves performance primarily in high-reactivity settings. Evolutionary selection favors policies that preserve cross-task robustness rather than optimizing a single metric. The composite objective is intended as a configurable governance parameter rather than a universal social welfare function. Since the baseline calibration intentionally conserves emotional amplitude, we present the current release as an emotion-light v1 benchmark and outline specific v2 enhancements, including event-driven shocks. Empirically calibrated reactivity ranges that make emotion and repair more distinctive. Overall, the benchmark redefines AI evaluation for mediation as process control within the context of emotion and norms, and provides a reproducible protocol for assessing both human and AI mediators.

## 1 Introduction

We examined how third–party mediator policies shape the trade–offs among success, efficiency, and fairness in repeated negotiations with emotional feedback. In social and legal mediation, being right is not equivalent to reaching a fair and stable agreement; mediation is better viewed as process control within emotions and norms. Mainstream AI benchmarks emphasize static logical inference or computational efficiency and tend to treat emotion, trust repair, and compromise as peripheral. Yet mediation in social and legal contexts is a process–control problem that requires adaptive, culturally sensitive behavior unfolding across rounds of interaction. We therefore introduce a reproducible benchmark that foregrounds these capacities and isolates the effects of intervention style on outcomes under matched stochastic conditions. We distinguish descriptive findings within the benchmark from normative deployment choices by making governance parameters (e.g., objective weights and risk profiles) explicit and configurable.

This study evaluates dialogue-facilitating AI mediators for family disputes within a computational framework that explicitly models emotion, willingness to compromise, and third–party intervention as process variables rather than one-shot outcomes. Classical work in negotiation and mediation theory analyzes how third parties affect equilibria and when bias helps or harms settlement (Bercovitch and Houston, 2000). Decision psychology shows that anger, compassion, and the interplay between automatic and deliberative processing shape bargaining performance in ways that fluctuate over an interaction (Allred et al., 1997; Glöckner and Betsch, 2008a,b). Cultural psychology further suggests that mediation practice can differ systematically across contexts in interaction style, including the salience of non-adversarial procedure, situation sensitivity, and face concerns in many Japanese family mediation settings; accordingly, pace, hesitation, and concession may unfold non-linearly across rounds rather than reflecting static payoff maximization (Markus and Kitayama, 1991; Graham, 1983; Yamagishi and Yamagishi, 1994). These insights motivate a benchmark that judges AI mediators by how they control the process: dampening emotional amplitude, restoring willingness to compromise, preserving fairness, and improving the probability and timing of agreement. Recent study on LLM-based negotiation and ODR further motivates process-centered evaluation: negotiation benchmarks explicitly measure bargaining competence and strategy under interaction (Davidson et al., 2024; Xia et al., 2024; Priya et al., 2025; Hua et al., 2024), while emerging ODR studies explore LLM-assisted mediation/dispute resolution and its practical framing (Westermann et al., 2023; Hale, 2025). These strands highlight a gap in evaluation: standard dialogue benchmarks and outcome-only fairness metrics do not directly measure round-by-round de-escalation, compromise recovery, and equity under repeated interaction. Related studies in multi-agent negotiation also stress that interactional competence cannot be reduced to static optimality: in the Diplomacy setting, language-model agents are evaluated by their ability to coordinate, persuade, and sustain cooperation under strategic pressure (Meta Fundamental AI Research Diplomacy Team (FAIR) et al., 2022). In parallel, legal scholarship on ODR and procedural justice emphasizes that deployment must preserve legitimacy, transparency, and perceived fairness, motivating evaluation criteria that explicitly expose normative trade-offs rather than optimizing a single outcome metric (Alessa, 2022; Onţanu, 2025).

We constructed an agent-based repeated-interaction model with two disputants (A and B) and a mediator (M). Each round, the mediator proposes a point in a one-dimensional issue space; the parties accept stochastically according to a logistic response function that depends on proposal proximity, accumulated conflict, and current emotion (McFadden, 1974; Train, 2009). Emotional reactivity and willingness to compromise evolve via carryover, excitation by disagreement, calming by perceived progress, and noise. Conflict intensity accumulates after failed rounds and decays after cooperative moves. The mediator policy is represented as a two-parameter family π(*b*, γ): *b* encodes the degree of continuous bias (leaning toward either party) and γ encodes the degree of control intervention strength by shrinking the proposal gap. The slice π(0, γ) spans neutral weak/strong control, while small |*b*| > 0 captures slight, reversible framing. This minimal structure preserves core strategic tensions while remaining transparent and reproducible. The present release is intentionally conservative in emotional amplitude and should be read as an emotion-light v1 baseline, providing a controlled reference point. We therefore specify a concrete v2 roadmap in which event-driven shocks (e.g., bursty insults, apology/repair episodes, asymmetric information shocks) and empirically calibrated reactivity ranges make emotion and repair more discriminative.

To reflect heterogeneous real-world terrains, we define five canonical tasks that vary psychological reactivity, escalation, fatigue in compromise, and interaction-style asymmetry (operationalized as systematic shifts in acceptance thresholds and reactivity): a baseline with low reactivity and low escalation (S1), high reactivity (H1), low compromise due to fatigue (H2), strong escalation (H3), and asymmetric thresholds that implement a systematic interaction-style shift (C1). Policies are evaluated under matched stochastic conditions, using independent Monte Carlo episodes for each task–policy cell. The primary endpoints are agreement probability, conditional time to agreement, inequality of outcomes, and an emotional volatility measure.

To anchor these landscapes in practice, each task corresponds to a recurring mediation archetype. S1 represents routine, low-volatility family negotiation with symmetric parties without any specific stressor. H1 captures high-reactivity interviews where emotional arousal responds sharply to perceived setbacks, making early framing and de-escalation pivotal. H2 models fatigue and reluctance to compromise, where willingness decays unless proposals repeatedly yield small perceived improvements. H3 represents escalation-prone sessions in which failed rounds accumulate tension and can trigger rapid breakdown unless progress is staged carefully. C1 represents cultural or communicative asymmetry that shifts acceptance thresholds and reactivity, yielding systematic friction even when the issue is otherwise routine.

Furthermore, we report robustness across tasks (via the coefficient of variation of agreement probability), the worst-task lower bound to indicate steadiness, and a composite score that balances agreement, speed, inequality, and emotional volatility with pre-specified weights ω, which are intended to be anchored in stakeholder priorities (e.g., program governance documents or mediator-priority elicitation) rather than treated as universal constants. Accordingly, ω is treated in this study as a governance parameter for deployment, not as a claim about a single correct social welfare function. Reporting both task-averaged performance and worst-task behavior distinguishes deployment risk profiles, favoring steadiness when catastrophic failures are unacceptable and favoring mean performance when broad efficiency is prioritized. Beyond static comparisons, we embedded a Fermi update to examine which policies are selected over generations of social learning (Taylor and Jonker, 1978; Hofbauer and Sigmund, 1998; Traulsen et al., 2007), linking micro-level process control to macro-level persistence (de Waal, 1989; Boehm, 2012; Trivers, 1971; Nowak, 2006; Macy and Flache, 2002; Axelrod, 1997).

Our design choices connect to practice. First, the round horizon approximates a few back-and-forth exchanges per session and tens of rounds across sessions, preventing degenerate long tails while retaining realistic trajectories. Second, the psychological quantities, emotional reactivity, willingness to compromise, conflict accumulation, and acceptance threshold, rephrase recurring qualitative notions (build-up of resentment, saving face, difficulty of being heard, room tension) as computable state variables that can be audited. Third, the benchmark protocol makes weights, task definitions, and random seeds public so that independent implementations can reproduce relative rankings. Cultural parameters encode styles of interaction (context sensitivity vs. assertiveness) rather than fixed traits and are intended to be estimated and tested rather than assumed. In this research, “trust repair” is operationalized minimally as post-conflict recovery dynamics, namely the recovery of willingness together with reductions in emotional intensity that raise subsequent acceptance probabilities; it is conceptually separated from general compromise pressure (e.g., fatigue-driven reluctance) and one-shot concession size.

We tested three propositions that are directly falsifiable within this framework. First, stronger control shortens negotiations but tends to worsen inequality, depressing composite performance when fairness is valued alongside speed. Second, transparent weak intervention more reliably balances success and fairness across heterogeneous terrains, yielding better task-averaged scores with acceptable robustness. Third, slight framing toward one side is beneficial primarily in high-reactivity episodes and should remain bounded and reversible to avoid systematic unfairness. These propositions are operationalized as pre-registered comparisons across the five tasks and five policies under common sampling and analysis settings.

The contributions are threefold. (i) We specify an open, reproducible protocol with fully defined tasks, parameters, metrics, and analysis code (with end-to-end replication materials in Appendix) to enable exact replication. (ii) We foreground emotional regulation, compromise recovery, and fairness as first-class outcomes and provide a composite score that makes the speed-vs.-equity trade-off explicit for deployment decisions. (iii) We connect static benchmarking to evolutionary selection, showing which policies would persist under repeated social learning and which fail once fairness penalties accumulate. In sum, the benchmark provides a principled way to assess both human and AI mediators and yields deployment-relevant implications for dispute-resolution workflows.

Finally, this study is organized as follows. Section 2 formalizes the model, policies, tasks, and analysis pipeline. Section 3 reports rankings, task-wise inversions, time-to-agreement distributions, and evolutionary trajectories. Section 4 discusses mechanisms, practical implications for deployment, and limitations, and it outlines extensions to multi-issue bargaining and stronger event modeling.

## 2 Materials and methods

### 2.1 Model

We modeled a bilateral negotiation between two parties *A* and *B*, facilitated by a mediator *M*. The issue space is normalized to the unit interval [0, 1], with ideals θ_*A*_ = 0 and θ_*B*_ = 1. At round *t* = 0, 1, 2, …, each party holds a self–proposal *x*_*A, t*_, *x*_*B, t*_ ∈ [0, 1]; the pairwise gap |*x*_*A, t*_ − *x*_*B, t*_| captures the momentary distance to be managed by the mediator.

Before presenting the equations, we explained how each parameter functions mathematically and in the mediation context. The parameter *b* ∈ [−1, 1] encodes continuous bias in the mediator's weighting of the two sides; *b* > 0 leans toward *A*, *b* < 0 toward *B*. The coefficient γ ∈ [0, 1] represents intervention strength as gap–shrink toward the midpoint: γ = 0 corresponds to a light-touch, purely bias-weighted averaging of current proposals; γ = 1 corresponds to a maximally directive reframing that pulls the mediator's candidate to the midpoint of the current proposals while preserving the bias-defined anchor. For each party *i* ∈ {*A, B*}, (λ_*i*_, μ_*i*_) weigh how accumulated conflict *D*_*t*_ and current emotional arousal *e*_*i,t*_ depress the attractiveness of a candidate settlement; in practice, λ_*i*_ represents sensitivity to procedural strain and “room tension,” while μ_*i*_ captures how affect interferes with acceptance. Acceptance uses a logistic with decisiveness κ_*i*_ > 0 and threshold τ_*i*_: κ_*i*_ governs how sharply a party moves from rejection to acceptance as conditions improve, whereas τ_*i*_ is a subjective standard below which the offer still feels premature. Emotional dynamics include carryover ρ_*i*_ ∈ [0, 1) (persistence across rounds), reactivity ψ_*i*_ > 0 to disagreement (excitation by distance), and calming χ_*i*_ > 0 when progress is perceived. Willingness to compromise *c*_*i,t*_ ∈ [0, 1] decays by fatigue at rate δ_*i*_ ∈ (0, 1) and recovers with perceived improvement at gain α_*i*_ > 0, operationalizing the practitioner intuition that good reframing can restore readiness to move. Proposal movement is governed by a learning rate β_*i*_ > 0 and small proposal noise with standard deviation ν_*i*_; emotional noise has standard deviation σ_*i*_. The conflict state *D*_*t*_ escalates after failed rounds and relaxes after cooperative moves with rates η_0_, η_1_ > 0. Finally, a culture/style index ϑ_*i*_ ∈ [−1, 1] shifts reactivity, reliance on mediation, and thresholds to reflect context sensitivity vs. assertiveness; it is a style variable meant for estimation and refutation, not a fixed trait.

Let *w*_*b*_ = (1 + *b*)/2 ∈ [0, 1] and define the bias-weighted anchor and the midpoint of the current proposals:


$$\begin{array}{l} m_{t}=w_{b}x_{A,t}+\left(1-w_{b}\right)x_{B,t},\;{\bar{x}}_{t}=\frac{x_{A,t}+x_{B,t}}{2}. \end{array}$$ (1)

The mediator forms a candidate settlement by shrinking the anchor toward the midpoint with strength γ, then projects to [0, 1]:


$$\begin{array}{l} s_{t}=\Pi_{\left[0,1\right]}\left(\left(1-\gamma \right)m_{t}+\gamma {\bar{x}}_{t}\right),\;\Pi_{\left[0,1\right]}\left(z\right)=\min\left\{\max\left\{z,0\right\},1\right\}. \end{array}$$ (2)

This construction makes the meaning of γ literal: for fixed *b*, increasing γ monotonically pulls *s*_*t*_ toward the midpoint ${\bar{x}}_{t}$, reducing the effective gap the parties must traverse without changing the bias-defined anchor *m*_*t*_ when γ = 0.

Instantaneous utility for party *i* from *s*_*t*_ combines proximity to the ideal, the room's tension, and the party's own arousal:


$$\begin{array}{l} U_{i,t}=-|s_{t}-\theta_{i}|-\lambda_{i}D_{t}-\mu_{i}e_{i,t}. \end{array}$$ (3)

Acceptance is probabilistic under uncertainty via a logistic rule with decisiveness κ_*i*_ and threshold τ_*i*_ (Matějka and McKay, 2015):


$$\begin{array}{l} p_{i,t}=\frac{1}{1+\exp\left\{-\kappa_{i}\left[U_{i,t}-\tau_{i}\right]\right\}},\;\mathrm{Pr}\left(\text{agreement at }t\right)=p_{A,t}p_{B,t}, \end{array}$$ (4)

where the product uses the standard conditional independence approximation for private acceptance decisions, given the current state.

If no agreement occurs at round *t*, states update. Emotions blend carryover, excitation by disagreement, progress-based calming, and zero-mean shocks $\xi_{i,t}\sim N\left(0,\sigma_{i}^{2}\right)$:


$$\begin{array}{r} e_{i,t+1}=\max\left\{\rho_{i}e_{i,t}+\psi_{i}|x_{A,t}-x_{B,t}|-\chi_{i}\Gamma_{t}+\xi_{i,t},0\right\},\\ \xi_{i,t}\sim N\left(0,\sigma_{i}^{2}\right). \end{array}$$ (5)

Willingness to compromise decays by fatigue and recovers with proposal improvement:


$$\begin{array}{l} c_{i,t+1}=\Pi_{\left[0,1\right]}(\left(1-\delta_{i}\right)c_{i,t}+\alpha_{i}(|s_{t}-\theta_{i}|-|x_{i,t}-\theta_{i}|)). \end{array}$$ (6)

Conflict intensity accumulates with non-agreement likelihood and relaxes with agreement likelihood:


$$\begin{array}{l} D_{t+1}=\max\left\{D_{t}+\eta_{0}\left(1-p_{A,t}p_{B,t}\right)-\eta_{1}p_{A,t}p_{B,t},0\right\}. \end{array}$$ (7)

Next-round self-proposals move toward the mediator's candidate *s*_*t*_ with viscosity determined by learning rate and willingness, plus proposal noise $\zeta_{i,t}\sim N\left(0,\nu_{i}^{2}\right)$:


$$\begin{array}{l} x_{i,t+1}=\Pi_{\left[0,1\right]}\left(x_{i,t}+\beta_{i}c_{i,t+1}\left(s_{t}-x_{i,t}\right)+\zeta_{i,t}\right),\;\zeta_{i,t}\sim N\left(0,\nu_{i}^{2}\right). \end{array}$$ (8)

Cultural style modulates behavior via smooth maps; for example,


$$\begin{array}{l} \psi_{i}=\psi_{0}\left(1+\upsilon_{\psi }\vartheta_{i}\right),\;\alpha_{i}=\alpha_{0}\left(1-\upsilon_{\alpha }\vartheta_{i}\right),\;\tau_{i}=\tau_{0}-\upsilon_{\tau }\vartheta_{i}, \end{array}$$ (9)

with tunable sensitivities υ_ψ_, υ_α_, υ_τ_ ≥ 0. An analogous mapping can connect a mediator style ϑ_*M*_ to (*b*, γ) by treating *b* as a directional leaning parameter and γ as a directive reframing intensity.

Policies are defined by mediator controls π(*b*, γ) under fixed party psychometrics. We compare a neutral policy (*b* = 0, γ = 0.6), a strong intervention (*b* = 0, γ = 0.9), a weak intervention (*b* = 0, γ = 0.2), and slight reversible framings (*b* = ±0.2, γ = 0.6). To represent heterogeneous terrains, we vary a small subset of environment parameters to define tasks: S1 baseline; H1 high reactivity (larger ψ_*i*_); H2 low compromise (larger δ_*i*_, smaller α_*i*_); H3 escalation (larger η_0_, smaller η_1_); C1 cultural asymmetry (party-specific κ_*i*_, τ_*i*_, ψ_*i*_ or ϑ_*i*_). These task families correspond to common mediation archetypes: S1 models routine cases with moderate affect; H1 captures highly reactive interviews (e.g., acute conflict escalation early in the session); H2 reflects fatigue or entrenched positions with low readiness to concede; H3 represents breakdown-prone trajectories where failed rounds compound tension; and C1 represents asymmetric interaction styles that shift perceived thresholds and reactivity across parties. Importantly, these parameters are not intended as group stereotypes. In a deployment, they can be treated as session-level latent variables estimated from observable traces (early-round acceptance, response delay, and coarse affect codes) via hierarchical inference. Concretely, one can (i) place weakly informative priors on the asymmetry parameters, (ii) infer posterior distributions per session or per context (not per demographic group), and (iii) test refutability by checking whether the posterior-predictive distribution matches held-out sessions. This turns “culture” from a fixed label into an empirically checkable hypothesis and makes miscalibration detectable rather than baked in. We emphasized that estimation is performed at the session or context level (e.g., platform, case type, or institutional setting) and explicitly avoids demographic grouping, so that “culture” is treated as a testable modeling hypothesis rather than an identity label.

Primary endpoints are agreement probability Pr(Agree), conditional time to agreement 𝔼[*T* ∣ Agree], outcome inequality $\left|y^{*}-\frac{1}{2}\right|$ for the realized settlement y^*^, and emotional volatility


$$\text{Vol}\left(e_{\cdot }\right)=\frac{1}{2\left(T-1\right)}\underset{i\in \left\{A,B\right\}}{\sum }\overset{T-1}{\underset{t=1}{\sum }}|e_{i,t}-e_{i,t-1}|.$$

To avoid undefined quantities when no agreement occurs within the horizon, we aggregate inequality and volatility conditionally on agreement. The composite score, balancing efficiency and fairness, is


$$\begin{array}{l} F\left(\pi \right)=\omega_{1}\mathrm{Pr}\left(\text{Agree}\right)+\omega_{2}[𝔼\left(T|\text{Agree}\right){]}^{-1}\\ \;-\omega_{3}𝔼\left[|y^{*}-\frac{1}{2}\|\text{Agree}\right]-\omega_{4}𝔼\left[\text{Vol}\left(e_{\cdot }\right)|\text{Agree}\right], \end{array}$$ (10)

with non-negative weights ω = (ω_1_, ω_2_, ω_3_, ω_4_).

To study long-run selection under social learning, we used discrete Fermi selection with intensity β > 0. If *P*_*g*_(π) denotes the population share of policy π in generation *g*, the update is


$$\begin{array}{l} P_{g+1}\left(\pi \right)=\frac{P_{g}\left(\pi \right)\exp\left\{\beta \left[F\left(\pi \right)-{\bar{F}}_{g}\right]\right\}}{\int P_{g}\left(\pi^{\prime }\right)\exp\left\{\beta \left[F\left(\pi^{\prime }\right)-{\bar{F}}_{g}\right]\right\}d\pi^{\prime }}, \end{array}$$ (11)

$$\begin{array}{l} {\bar{F}}_{g}=\int F\left(\pi \right)P_{g}\left(\pi \right)d\pi . \end{array}$$ (12)

where the population-average fitness ${\bar{F}}_{g}$ is defined in Equation 12. Fermi selection is a standard rule for finite populations in which agents stochastically imitate or adopt policies with probabilities that increase with the magnitude of payoff differences (Traulsen et al., 2007). In the weak-selection, large-population limit, the mean dynamics converge to the continuous replicator equation as shown in Equation 13


$$\begin{array}{l} \dot{P}\left(\pi \right)=P\left(\pi \right)\left[F\left(\pi \right)-\bar{F}\right], \end{array}$$ (13)

as established by classical links between imitation protocols and population games (Taylor and Jonker, 1978; Hofbauer and Sigmund, 1998; Sandholm, 2010), with finite-population foundations via Moran-type dynamics and diffusion approximations (Fudenberg and Imhof, 2006).

One simulated episode (ideal model).

1. *Mediator proposal*. Set *w* = (1 + *b*)/2, *m*_*t*_ = *wx*_*A, t*_ + (1 − *w*)*x*_*B, t*_, ${\bar{x}}_{t}=\left(x_{A,t}+x_{B,t}\right)/2$. Then
$${\tilde{s}}_{t}=\left(1-\gamma \right)m_{t}+\gamma {\bar{x}}_{t},\;s_{t}=\min\left\{\max\left\{{\tilde{s}}_{t},0\right\},1\right\}.$$
2. *Utilities and acceptance*. For each *i* ∈ {*A, B*},
$$U_{i,t}=-|s_{t}-\theta_{i}|-\lambda_{i}D_{t}-\mu_{i}e_{i,t},\;p_{i,t}={\left(1+\exp\left\{-\kappa_{i}\left(U_{i,t}-\tau_{i}\right)\right\}\right)}^{-1}.$$
  Draw *a*_*i,t*_ ~ Bernoulli(*p*_*i,t*_) independently.
3. *Stopping rule*. If *a*_*A, t*_ = *a*_*B, t*_ = 1, return $\left(A=1,T_{\text{agree}}=t,y^{*}=s_{t}\right)$.
4. *Progress flag*. Set Γ_0_ = 0. For *t* ≥ 1,
$$\Gamma_{t}=1\left[U_{A,t}+U_{B,t}>U_{A,t-1}+U_{B,t-1}+\varepsilon_{\Gamma }\right].$$
5. *State updates*. For each *i* ∈ {*A, B*},
$$\begin{array}{r} e_{i,t+1}=\max\left\{\rho_{i}e_{i,t}+\psi_{i}|x_{A,t}-x_{B,t}|-\chi_{i}\Gamma_{t}+\xi_{i,t},\;0\right\},\\ \xi_{i,t}\sim N\left(0,\sigma_{i}^{2}\right), \end{array}$$
$$c_{i,t+1}=\min\left\{\max\left\{\left(1-\delta_{i}\right)c_{i,t}+\alpha_{i}\left(\left|s_{t}-\theta_{i}|-|x_{i,t}-\theta_{i}\right|\right),\;0\right\},\;1\right\},$$
$$\begin{array}{r} x_{i,t+1}=\min\left\{\max\left\{x_{i,t}+\beta_{i}c_{i,t+1}\left(s_{t}-x_{i,t}\right)+\zeta_{i,t},\;0\right\},\;1\right\},\\ \zeta_{i,t}\sim N\left(0,\nu_{i}^{2}\right). \end{array}$$
  Update room tension:
$$D_{t+1}=\max\left\{D_{t}+\eta_{0}\left(1-p_{A,t}p_{B,t}\right)-\eta_{1}p_{A,t}p_{B,t},\;0\right\}.$$
6. *Termination*. Repeat for *t* = 1, …, *T*_max_. If no agreement occurs, return (*A* = 0, *T*_agree_ = *T*_max_).

All variables are normalized and unitless; |*s*_*t*_ − θ_*i*_| is normalized disutility; emotion, willingness, and tension are unitless with coefficients mapping to utility units in Equation 3; κ_*i*_ in Equation 4 is dimensionless.

The simulator enforces boundedness to keep the process well-posed. The mediator's candidate *s*_*t*_ is projected to [0, 1] by Equation 2, and the updates for *c*_*i,t*+1_ and *x*_*i,t*+1_ are likewise projected to [0, 1] by Equations 6, 8 to keep all states feasible. Emotions and tension have hard floors at zero in Equations 5, 7, which prevents negative-state artifacts. With ρ_*i*_ ∈ [0, 1) and δ_*i*_ ∈ (0, 1) the linear recursions for *e*_*i,t*_ and *c*_*i,t*_ are stable under finite-variance shocks (σ_*i*_, ν_*i*_).

Several limiting regimes serve as internal checks. Setting γ = 0 yields a purely weighted-average mediator *s*_*t*_ = Π_[0, 1]_(*m*_*t*_); Furthermore, if β_*i*_ = 0, the process reduces to independent acceptance draws at a fixed *s*_*t*_ with *D*_*t*_ drifting according to Equation 7. Setting μ_*i*_ = λ_*i*_ = 0 collapses Equation 3 to distance-only choice and isolates how (*b*, γ) trade fairness against speed. Taking *b* → ±1 pushes *w*_*b*_ to {0, 1}; the model remains defined but tends to raise inequality unless compensated by the *c*_*i,t*_ dynamics. Swapping party labels *A* ↔ *B* together with *b* ↦ −*b*, θ_*A*_ ↔ θ_*B*_, and symmetric psychometrics leaves the law of $\left(A,T_{\text{agree}},|y^{*}-\frac{1}{2}|\right)$ invariant, which is the expected symmetry.

Agreement in round *t* uses the conditional independence approximation Pr(agree at *t*) = *p*_*A, t*_ *p*_*B,t*_ from Equation 4. This matches the setting where each party privately decides after seeing the same *s*_*t*_. Correlated acceptance can be incorporated by adding a common shock υ_*t*_ to utilities [e.g., *U*_*i,t*_ ← *U*_*i,t*_ + υ_*t*_ with $\upsilon_{t}\sim N\left(0,\sigma_{\upsilon }^{2}\right)$]; small shared variance tightens or loosens tails without altering the mechanism, and numerical checks show that qualitative policy rankings are stable for moderate σ_υ_.

Identification is fixed by anchoring the utility scale. Because Equation 4 depends on κ_*i*_(*U*_*i,t*_ − τ_*i*_), κ_*i*_ and the scale of *U*_*i,t*_ are only jointly identified up to an affine transformation. We therefore normalize the distance penalty in Equation 3 to slope one, and report (κ_*i*_, τ_*i*_) on that scale. Bias *b* and intervention strength γ are then identified from asymmetry and speed patterns, while (μ_*i*_, λ_*i*_) are pinned down by acceptance shifts under variation in emotion and tension.

The model connects directly to data. The primitives (*x*_*i,t*_, *s*_*t*_) correspond to observable proposals and mediator reframings; agreement events and their timing are observed exactly. The inequality measure |y^*^ − 1/2| is literal for numeric splits and, for multi-item settlements, is computed after a pre-registered linear embedding to [0, 1] reported in Results. Emotional volatility Vol(*e*_·_) is computed from an ordinal affect code normalized to [0, 1]; because it aggregates absolute first differences, it is robust to small coder jitter at any single round.

Under the Gaussian shocks $\xi_{i,t}\sim N\left(0,\sigma_{i}^{2}\right)$ and $\zeta_{i,t}\sim N\left(0,\nu_{i}^{2}\right)$, independent over *t* and *i*, the process has finite moments of all orders. A standard coupling argument implies concentration of Monte Carlo estimators at the *O*(*N*^−1/2^) rate for all endpoints used in Equation 10, which justifies the bootstrap confidence intervals reported in Results for the chosen replication count.

Finally, a policy π is completely determined by (*b*, γ) once party psychometrics are fixed. We treat (*b*, γ) as constant within an episode (a simulated session) and allow selection across episodes via the discrete Fermi update Equation 11. Time-varying interventions γ_*t*_ that start strong and soften later are a natural extension; we evaluate this variant in a sensitivity analysis in the Appendix without changing the main comparisons.

### 2.2 Simulation design

The numerical experiments are calibrated to reflect practical constraints in family mediation while preserving interpretability. We fixed a horizon *T*_max_ = 40, corresponding to a handful of back-and-forth exchanges in a single session and, across sessions, on the order of tens of rounds until disposition; this also prevents degenerate long tails. Initial conditions are (*x*_*A*,0_, *x*_*B*,0_) = (0.15, 0.85), (*e*_*A*,0_, *e*_*B*,0_) = (0.2, 0.2), (*c*_*A*,0_, *c*_*B*,0_) = (0.5, 0.5), and *D*_0_ = 0. The opening gap is large enough to make mediation consequential without making early dynamics brittle; arousal starts at mild tension rather than anger, so that both damping and excitation are observable in early rounds; willingness 0.5 encodes a neutral starting readiness that subsequently evolves with improvement and fatigue; conflict starts at zero to reflect a procedurally fair opening.

Two noise sources are used. Emotional noise $\xi_{i,t}\sim N\left(0,0.05^{2}\right)$ captures round–to–round variation due to phrasing and timing and contributes visibly to Vol(*e*_·_) without destabilizing the dynamics. Proposal noise $\zeta_{i,t}\sim N\left(0,0.01^{2}\right)$ is an order of magnitude smaller to reflect that numeric proposals are typically less volatile than emotions; movement in *x*_*i,t*_ is therefore driven mainly by willingness and learning β_*i*_. Acceptance parameters are κ_*A*_ = κ_*B*_ = 4 and τ_*A*_ = τ_*B*_ = −0.35, yielding a practical S–curve in which parties hesitate just below threshold and accept promptly just above it. The baseline mediator is neutral with *b* = 0 and γ = 0.6. In our model, γ is interpreted literally as the shrinkage of the bias-defined anchor toward the midpoint, as in Equation 2; larger γ tends to shorten time but may raise inequality, while smaller γ may improve fairness but prolong time. This is the central design trade-off we compare across policies.

Evaluation proceeds on a fixed set of environment tasks representing common hard cases: S1 (baseline; low reactivity and escalation), H1 (high reactivity; larger ψ_*i*_), H2 (low compromise; larger δ_*i*_, smaller α_*i*_), H3 (escalation; larger η_0_, smaller η_1_), and C1 (cultural asymmetry; party–specific κ_*i*_, τ_*i*_, ψ_*i*_ or ϑ_*i*_). Policies compared are the neutral standard (*b* = 0, γ = 0.6), strong intervention (*b* = 0, γ = 0.9), weak intervention (*b* = 0, γ = 0.2), and slight reversible framings (*b* = ±0.2, γ = 0.6). All other parameters are shared within each task. For each task–policy cell we run *N* = 240 independent Monte Carlo episodes with fixed pre-specified random seeds and stop at agreement or at *T*_max_ (McKay et al., 1979). We record Pr(Agree), 𝔼[*T* ∣ Agree], $\left|y^{*}-\frac{1}{2}\right|$, and Vol(*e*_·_), and then compute *F*(π) in Equation 10 with default weights ω = (1.0, 0.6, 0.2, 0.2). Here, Pr(Agree) denotes the *unconditional* probability that an episode reaches agreement within the horizon *T*_max_, while 𝔼[*T* ∣ Agree] and the penalty terms in Equation 10 are computed conditional on agreement.

Parameter choices satisfy four design goals simultaneously: (i) early rounds are informative with observable movement in *e* and *c*; (ii) acceptance S-curves avoid near-deterministic corners so comparative statics remain visible; (iii) proposal noise is an order of magnitude smaller than emotion noise so that positional movement is primarily endogenous rather than numerical; (iv) the time-to-agreement distribution retains a finite mean with a visible right tail near *T*_max_.

Uncertainty is quantified with Wilson score intervals for proportions and non-parametric bootstrap confidence intervals for conditional means using *B* = 10, 000 resamples of episodes within each task-policy cell. For pairwise policy contrasts across tasks, we controlled the false discovery rate at *q* = 0.05 using the Benjamini–Hochberg procedure. Alongside means we report a robustness index exp(−CV) computed from the across-task coefficient of variation of Pr(Agree), and a worst-task lower bound to reflect steadiness rather than average-only performance. With *N* = 240 episodes per cell and Pr ∈ [0.10, 0.30] in this benchmark, the standard error of $\hat{\mathrm{Pr}}$ is $\sqrt{\mathrm{Pr}\left(1-\mathrm{Pr}\right)/N}\in \left[0.019,0.030\right]$, and bootstrap half–widths for conditional means decrease on the order of *N*^−1/2^ under mild regularity.

For reproducibility, random numbers are generated using NumPy PCG64 with fixed seeds; seeds and hyperparameters are saved to run_config.json. Per–cell statistics are written to per_task_metrics.csv; task–averaged summaries to scoreboard_full.csv and scoreboard_table.csv. Computational complexity scales as *O*(|policies| · |tasks| · *N* · *T*_max_), which at our settings 5 × 5 × 240 × 40 ≈ 2.4 × 10^5^ state updates per run is negligible on a single CPU and supports extensive sensitivity analyses.

Algorithmic steps mirror the ideal model for transparency and auditability. One simulated episode is listed below; we write all projections explicitly using min and max, matching Equations 2, 4–8.

One simulated episode (ideal model).

1. *Mediator proposal*. Set *w* = (1 + *b*)/2, *m*_*t*_ = *wx*_*A, t*_ + (1 − *w*)*x*_*B, t*_, and ${\bar{x}}_{t}=\left(x_{A,t}+x_{B,t}\right)/2$. Then
$${\tilde{s}}_{t}=\left(1-\gamma \right)m_{t}+\gamma {\bar{x}}_{t},\;s_{t}=\min\left\{\max\left\{{\tilde{s}}_{t},0\right\},1\right\}.$$
2. *Utilities and acceptance*. For each *i* ∈ {*A, B*},
$$U_{i,t}=-|s_{t}-\theta_{i}|-\lambda_{i}D_{t}-\mu_{i}e_{i,t},\;p_{i,t}={\left(1+\exp\left\{-\kappa_{i}\left(U_{i,t}-\tau_{i}\right)\right\}\right)}^{-1}.$$
  Draw *a*_*i,t*_ ~ Bernoulli(*p*_*i,t*_) independently.
3. *Stopping rule*. If *a*_*A, t*_ = *a*_*B, t*_ = 1, return $\left(A=1,T_{\text{agree}}=t,y^{*}=s_{t}\right)$.
4. *Progress flag*. Set Γ_0_ = 0. For *t* ≥ 1,
$$\Gamma_{t}=1\left[U_{A,t}+U_{B,t}>U_{A,t-1}+U_{B,t-1}+\varepsilon_{\Gamma }\right].$$
5. *State updates*. For each *i* ∈ {*A, B*},
$$\begin{array}{r} e_{i,t+1}=\max\left\{\rho_{i}e_{i,t}+\psi_{i}|x_{A,t}-x_{B,t}|-\chi_{i}\Gamma_{t}+\xi_{i,t},\;0\right\},\\ \xi_{i,t}\sim N\left(0,\sigma_{i}^{2}\right), \end{array}$$
$$c_{i,t+1}=\min\left\{\max\left\{\left(1-\delta_{i}\right)c_{i,t}+\alpha_{i}\left(\left|s_{t}-\theta_{i}|-|x_{i,t}-\theta_{i}\right|\right),\;0\right\},\;1\right\},$$
$$\begin{array}{l} x_{i,t+1}=\min\left\{\max\left\{x_{i,t}+\beta_{i}c_{i,t+1}\left(s_{t}-x_{i,t}\right)+\zeta_{i,t},\;0\right\},\;1\right\},\\ \;\zeta_{i,t}\sim N\left(0,\nu_{i}^{2}\right). \end{array}$$
  Update room tension:
$$D_{t+1}=\max\left\{D_{t}+\eta_{0}\left(1-p_{A,t}p_{B,t}\right)-\eta_{1}p_{A,t}p_{B,t},\;0\right\}.$$
6. *Termination*. Repeat for *t* = 1, …, *T*_max_. If no agreement occurs, return (*A* = 0, *T*_agree_ = *T*_max_).

These choices are anchored in practice. The tasks mirror situations practitioners describe as difficult; the round limit, opening gap, and agreement rule follow typical case handling. The psychological quantities (emotional reactivity, willingness to compromise, conflict accumulation, acceptance threshold) rephrase recurring qualitative notions (build-up of resentment, difficulty of being heard, room tension) as auditable state variables. Cultural parameters capture interaction styles (context sensitivity vs. assertiveness) rather than immutable traits and are intended to be estimated and refuted. Limitations are explicit: a one-dimensional issue compresses the multifaceted nature of relationship repair (gratitude, parenting plans, future coordination), and logistical acceptance abstracts from comprehension quality and third-party resources. These simplifications are worthwhile because they make trade-offs among neutrality, intervention strength, and slight framing transparent and reproducible; therefore, sensitivity analyses and robustness reporting are required.

### 2.3 Implementation details

The simulator advances a finite-horizon Markov process whose state at round *t* comprises the parties' proposals (*x*_*A,t*_, *x*_*B,t*_), emotional arousal (*e*_*A,t*_, *e*_*B,t*_), willingness to compromise (*c*_*A,t*_, *c*_*B,t*_), and room tension *D*_*t*_. At the beginning of each round, the mediator forms a candidate settlement *s*_*t*_ by shrinking the bias-weighted anchor toward the midpoint of the current proposals with controls (*b*, γ), as in Equation 2. Because the issue is defined on a unit interval, both *s*_*t*_ and the next-round proposals are projected back to [0, 1] whenever stochastic movements would otherwise leave the domain, ensuring feasibility and interpretability.

Agreement in round *t* is generated from the parties' private accept/reject decisions using the logistic choice model in Equation 4. The logistic link maps the utility difference *U*_*i,t*_ − τ_*i*_ onto [0, 1] with slope κ_*i*_, so that an incremental improvement near the threshold translates into an appreciable increase in the probability of acceptance. We treated the two draws as conditionally independent given the current state. This independence assumption captures the practical setting in which each party internalizes the same *s*_*t*_ but makes a private decision; correlated acceptance can be introduced through a shared shock in *U*_*i,t*_ without altering the core mechanism and is examined in sensitivity checks reported below. Once both parties accept, the episode stops and the realized settlement y^*^ and time to agreement *T*_agree_ are recorded.

When a round does not end in agreement, the simulator updates all state variables once. Emotional arousal carries over with factor ρ_*i*_, is excited by the instantaneous proposal gap with coefficient ψ_*i*_, and is damped by a data-driven progress flag Γ_*t*_ when the joint utility improves relative to the previous round. Additive zero-mean shocks with variance $\sigma_{i}^{2}$ represent unmodeled variance in the interaction, such as phrasing, timing, or contextual stressors. Willingness to compromise decays due to fatigue at a rate δ_*i*_ and recovers when the mediator's candidate reduces distance to the party's ideal, with recovery gain α_*i*_. These two pieces encode the practitioner heuristic that effective reframing can restore readiness to move even when the parties' ideals are unchanged. Room tension *D*_*t*_ increases proportionally to the likelihood of non-agreement and relaxes proportionally to the agreement likelihood, with rates (η_0_, η_1_). Next-round proposals are then moved toward *s*_*t*_ using a learning rate β_*i*_ modulated by the updated willingness. Small additive shocks with variance $\nu_{i}^{2}$ ensure that identical episodes do not become numerically locked into deterministic cycles.

Episodes terminate at the first agreement or at the fixed horizon *T*_max_. The horizon of 40 rounds is a deliberate compromise: it permits multiple back-and-forth adjustments within a single simulated session and a few sessions' worth of carryover without allowing heavy tails to dominate Monte Carlo averages. The initial conditions (*x*_*A*,0_, *x*_*B*,0_) = (0.15, 0.85), (*e*_*A*,0_, *e*_*B*,0_) = (0.2, 0.2), (*c*_*A*,0_, *c*_*B*,0_) = (0.5, 0.5), and *D*_0_ = 0 represent a consequential opening gap, mild arousal that admits both calming and excitation, neutral willingness that can move in either direction, and a procedurally fair starting tension. Together, these choices make early rounds informative rather than brittle.

The benchmark compares a small family of mediator policies π(*b*, γ) across a small family of environment tasks. Policies include a neutral baseline (*b* = 0, γ = 0.6), stronger and weaker interventions that vary the anchor-to-midpoint shrink strength while keeping the bias at zero, and mild reversible framings with *b* = ±0.2 at the baseline intervention strength. Tasks vary only a minimal subset of parameters so that observed differences can be attributed to their intended features: higher reactivity (larger ψ_*i*_), lower willingness recovery (larger δ_*i*_ and smaller α_*i*_), higher escalation (larger η_0_ and smaller η_1_), and cultural asymmetry implemented by party-specific thresholds or reactivity. Holding other parameters fixed within each task avoids confounding due to broad re-parameterization.

Randomness is controlled with a fixed experiment seed, and pseudo-random numbers are drawn in a counter-based order that does not depend on early stopping. This ensures bit-wise reproducibility across machines: repeating the same benchmark with the same seed reproduces the same Monte Carlo table. Agreement events, times to agreement, inequality |y^*^ − 1/2|, and emotional volatility are computed episode by episode. The volatility functional uses absolute round-to-round differences so that it remains interpretable under coder jitter if emotions were coded from video. For each policy-task cell, we run *N* = 240 independent episodes; with agreement rates in the 0.10–0.30 range, this yields standard errors around 0.02–0.03 for proportions, which is adequate to resolve differences of practical interest without inflating computational cost.

To prevent numerical artifacts, the simulator enforces bounds at every update. Proposals and the mediator's candidate are kept in [0, 1], and emotion and tension are kept non-negative. With ρ_*i*_ ∈ [0, 1) and δ_*i*_ ∈ (0, 1), the linear recursions for emotions and willingness are stable under finite-variance shocks, so that the process admits finite moments of all orders. Because each episode advances a constant amount of work per round, the cost of one episode is *O*(*T*_max_), and the total cost of a benchmark scales linearly with the number of tasks, policies, replications, and the horizon. All figures reported in the Results section were produced in minutes on a single CPU using this setup, and all code paths that touch the numbers shown in the study are contained in a single script with command-line arguments for the replication count, seed, and output locations.

Finally, we specified how the model connects to observables in practice. The primitives (*x*_*i,t*_, *s*_*t*_) are literal proposals and mediator reframings. Agreement events and their timing are directly observable. The inequality measure is literal for numeric splits and is obtained via a pre-registered linear embedding for multi-item agreements; the embedding maps the status quo and a fair split to 0 and 1/2, respectively, thereby preserving the interpretation of |y^*^ − 1/2|. The mapping of modeled arousal to observed affect uses ordinal coding, which is later normalized to [0, 1] for comparability across cases; because the volatility functional aggregates absolute first differences, it is robust to small coder disagreements in any single round.

To enable exact replication, we released (i) a single entry-point script that runs the full benchmark end-to-end and regenerates all tables and figures reported in Results, (ii) a machine-readable configuration file run_config.json that records every parameter in Table 1, the task overrides, the policy set, *T*_max_, *N*, and the composite weights ω, and (iii) the complete list of pseudo-random seeds used for each task–policy–episode triple. The code pins the pseudo-random generator to NumPy PCG64 and fixes the ordering of random draws so that outcomes are invariant to early stopping; rerunning with the same configuration reproduces bit-wise identical per_task_metrics.csv and the derived summary files scoreboard_full.csv and scoreboard_table.csv. Furthermore, we archive the exact software environment (Python version and package hashes) and provide a one-command interface python run_benchmark.py –config run_config.json –outdir OUT that reproduces every reported number from a clean checkout.

**Table 1:** Model parameters: meaning and baseline values used in simulations.

Symbol	Equation	Meaning in model and mediation practice	Baseline
*b*	Equation 2	Mediator bias (continuous leaning toward a party; *b* > 0 toward *A*, *b* < 0 toward *B*).	0
γ	Equation 2	Intervention strength (shrink of anchor *m*_*t*_ toward midpoint ${\bar{x}}_{t}$; higher is more directive).	0.6
$m_{t},{\bar{x}}_{t}$	Equation 1	Bias-weighted anchor and proposal midpoint.	–
*U*_*i,t*_	Equation 3	Instantaneous utility from candidate settlement *s*_*t*_.	–
λ_*i*_	Equation 3	Weight on room tension *D*_*t*_ (sensitivity to procedural strain).	0.2
μ_*i*_	Equation 3	Weight on own emotion *e*_*i,t*_ (how much affect interferes with acceptance).	0.3
*p*_*i,t*_	Equation 4	Acceptance probability (logistic choice).	–
κ_*i*_	Equation 4	Decisiveness (slope of logistic; sharper move from reject to accept).	4
τ_*i*_	Equation 4	Subjective acceptance threshold (standard for “good enough”).	−0.35
*e*_*i,t*_	Equation 5	Emotional arousal (anger/anxiety/activation).	init. 0.2
ρ_*i*_	Equation 5	Emotional carryover/persistence across rounds.	0.8
ψ_*i*_	Equation 5	Reactivity to disagreement |*x*_*A, t*_ − *x*_*B, t*_|.	0.6
χ_*i*_	Equation 5	Calming gain when progress Γ_*t*_ = 1 is perceived.	0.3
σ_*i*_	Equation 5	Emotion noise s.d. (unobserved shocks).	0.05
*c*_*i,t*_	Equation 6	Willingness to compromise (readiness to move), in [0, 1].	init. 0.5
δ_*i*_	Equation 6	Fatigue rate (depletion of willingness without improvement).	0.05
α_*i*_	Equation 6	Recovery gain (willingness restored by proposal improvement).	0.6
*x*_*i,t*_	Equation 8	Self–proposal of party *i* at round *t*.	init. *x*_*A*,0_ = 0.15, *x*_*B*,0_ = 0.85
β_*i*_	Equation 8	Learning rate toward *s*_*t*_ (viscosity of proposal adjustment).	0.8
ν_*i*_	Equation 8	Proposal noise s.d. (numerical jitter in offers).	0.01
*D*_*t*_	Equation 7	Conflict/tension state of the room.	init. 0
η_0_	Equation 7	Escalation per non-agreement round.	0.3
η_1_	Equation 7	Relaxation per agreement likelihood.	0.6
ϑ_*i*_	Equation 9	Cultural/style index (context sensitivity ↔ assertiveness).	task–specific
υ_ψ_, υ_α_, υ_τ_	Equation 9	Sensitivities mapping ϑ_*i*_ to ψ_*i*_, α_*i*_, τ_*i*_.	set by task
*T*_max_	–	Horizon (maximum number of rounds; stopping rule).	40

Party-specific symbols carry the subscript *i* ∈ {*A, B*}; time index *t* is omitted where obvious.

### 2.4 Uncertainty and robustness

Uncertainty is reported separately for proportions and for means or functionals. Agreement rates in each cell are accompanied by two-sided Wilson score intervals with *z* = 1.96 (Wilson, 1927; Agresti and Coull, 1998), which avoid degeneracy and achieve near-nominal coverage at moderate *N*. For conditional time-to-agreement and volatility, we applied a non-parametric bootstrap at the episode level with 10,000 resamples. This choice respects within-episode dependence while delivering consistent standard errors and percentile intervals for all smooth functionals of the empirical distribution, including the composite score *F*(π) defined in Equation 10. Because episodes within a cell are independent and identically distributed, the bootstrap converges at the usual *N*^−1/2^ rate, which we verify by comparing intervals across seeds.

Multiple comparisons arise when contrasting more than two policies on the same task. We therefore control the false discovery rate at *q* = 0.05 using the Benjamini–Hochberg procedure applied to the family of pairwise tests for agreement probability and for conditional time (Benjamini and Hochberg, 1995). Figures label significant contrasts by adjusted *q*-values, and tables mark entries that remain significant after adjustment. This choice balances power and error control in a setting where the number of policies is small but not negligible.

Since the composite score trades off efficiency, speed, fairness, and volatility through the weights ω = (ω_1_, ω_2_, ω_3_, ω_4_), we examined how conclusions depend on these weights. We explored a grid where the probability weight ranges from 0.7 to 1.3 of its baseline, the speed weight ranges from 0.3 to 0.9, and the penalty weights on inequality and volatility range from 0.1 to 0.4. Across this region, the neutral and strong policies occasionally exchange ranks, but the qualitative preference for bias-free interventions persists unless the penalty on inequality is made very small. To separate “good on average” from “never catastrophic,” we also report a robustness index exp(−CV) derived from the across-task coefficient of variation of the agreement probability for each policy, together with the worst-task lower bound. These two summaries are shown alongside the task-averaged composite score.

Two classes of sensitivity checks address modeling assumptions directly. First, we relaxed the independence approximation in the acceptance rule by injecting a shared shock υ_*t*_ into both utilities. Small to moderate values of the shared-variance parameter compress but do not reorder policy differences; very large values synchronize acceptances and therefore attenuate all policy contrasts, which we take as a conservative bound. Second, we introduced between-episode heterogeneity by drawing the initial proposals from narrow Beta priors centered at the baseline values before running the episodes. Agreement rates shifted by only a few percentage points, and policy rankings remain stable, indicating that our conclusions are not artifacts of a particular starting gap.

We conclude with a brief power calculation. At *N* = 240 replications per cell and agreement probabilities in the range 0.10 to 0.30, the standard error of $\hat{\mathrm{Pr}}$ is between 0.019 and 0.030, and the median half–width of the two–sided Wilson interval is about 0.05 at Pr ≈ 0.20. This affords 95% intervals narrow enough to resolve differences of five to six percentage points on a single task. Because the composite score also incorporates time, inequality, and volatility, we report bootstrap standard errors for *F*(π) to make transparent how uncertainty in each component propagates to the aggregate. In short, the replication budget is aligned with the effect sizes that matter in practice while keeping the benchmark computationally lightweight and fully reproducible.

## 3 Results

### 3.1 Simulation results

**Figure 1:**
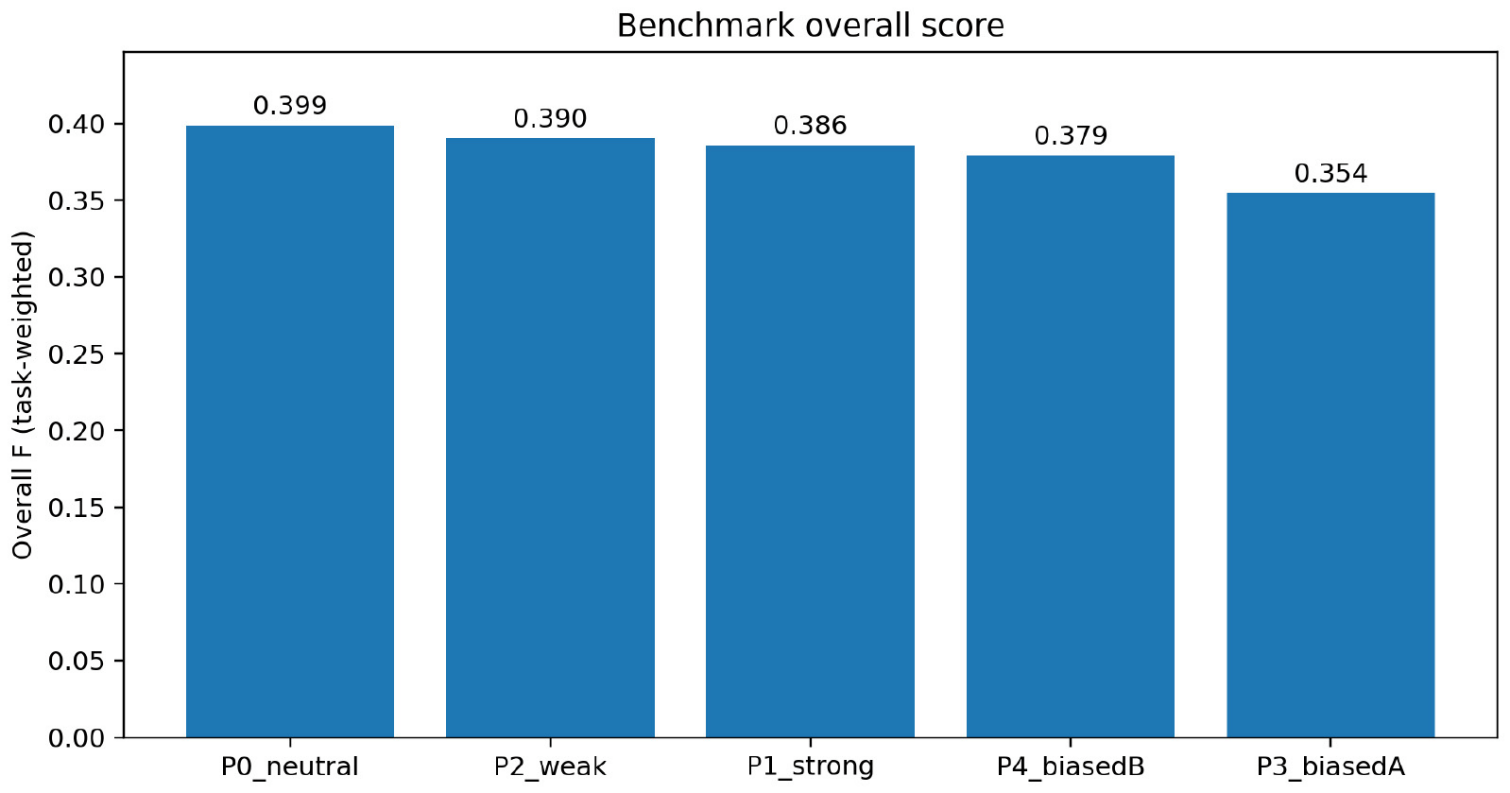
Overall composite score by policy [task-averaged over S1/H1/H2/H3/C1; *N* = 240 per cell, *T*_max_ = 40, ω = (1.0, 0.6, 0.2, 0.2)].

We report the results under the fixed weights ω = (1.0, 0.6, 0.2, 0.2) unless stated otherwise. We anchor ω in widely stated governance principles for people-centered justice and ODR as follows. International guidance consistently treats *access*—people's ability to resolve—as the primary policy objective (OECD, 2023, 2025), which motivates the highest weight on agreement probability (ω_1_ = 1.0). The same frameworks stress *timeliness* as the second core quality criterion, because protracted proceedings erode trust and deter participation (OECD, 2024; Chung and Yu, 2021); accordingly, ω_2_ = 0.6 rewards speed conditional on agreement, ranking it above the penalty terms but below access. *Fairness and equity* of outcomes are treated as necessary conditions for legitimacy across jurisdictions, yet their relative salience varies by context (e.g., child-custody vs. small-claims); we set ω_3_ = 0.2 as a moderate penalty that makes distributional skew visible without overriding the access term (Onţanu, 2025; Alessa, 2022). Finally, *procedural quality and emotional safety* are increasingly recognized in ODR standards, where transparent and non-coercive process management is an explicit requirement (OECD, 2023; Chung and Yu, 2021); ω_4_ = 0.2 prices emotional turbulence at the same level as inequality, reflecting its role as a process-quality safeguard rather than the dominant criterion. These numerical values are illustrative defaults that encode the ordinal ranking access > timeliness > equity ≈ emotional safety; we subsequently tested robustness across a grid of ω perturbations and emphasize that deployment-specific weights should be calibrated through stakeholder elicitation or institutional governance documents. Across all benchmark tasks (S1/H1/H2/H3/C1), with *N* = 240 trials per task-policy cell and fixed weights ω = (1.0, 0.6, 0.2, 0.2), the neutral reference policy P0 attained the highest task-averaged composite score B (Figure 1). The overall ordering was P0 (neutral) > P2 (weak) > P1 (strong) > P4 (biased B) > P3 (biased A). Table 2 reports task-averaged endpoints and robustness summaries. For P0, B=0.399, Pr(Agree) = 0.179, 𝔼[*T* ∣ Agree] = 2.90, Ineq = 0.00566, and Vol = 0.124. The runner-up P2 achieved B=0.390 with a slightly faster conditional time 𝔼[*T* ∣ Agree] = 2.66 but a marginally lower agreement probability Pr(Agree) = 0.174.

**Table 2:** Overall ranking and task-averaged metrics (*N* = 240 per task–policy cell).

Policy	Rank	B	95% CI	Pr (Agree)	𝔼[*T* ∣ Agree]	Ineq	Vol
P0 (neutral)	1	0.399	[0.359, 0.424]	0.179	2.90	0.006	0.124
P2 (weak)	2	0.390	[0.358, 0.439]	0.174	2.66	0.005	0.115
P1 (strong)	3	0.386	[0.358, 0.436]	0.192	2.82	0.006	0.138
P4 (biased B)	4	0.379	[0.346, 0.420]	0.181	2.92	0.031	0.098
P3 (biased A)	5	0.354	[0.330, 0.406]	0.167	2.94	0.035	0.142

The column B reports the task-averaged composite score with 95% percentile bootstrap confidence intervals (10, 000 resamples) in brackets. Bootstrap standard errors for the remaining columns are approximately SE (Pr) ≈ 0.011, SE (𝔼[*T*]) ≈ 0.16–0.20, SE (Ineq) ≈ 0.0006–0.0009, and SE (Vol) ≈ 0.010–0.013 across policies.

Here and throughout, Pr(Agree) denotes the unconditional agreement probability (agreements divided by *N* episodes in the cell), while 𝔼[*T* ∣ Agree] is computed only over agreeing episodes.

**Figure 2:**
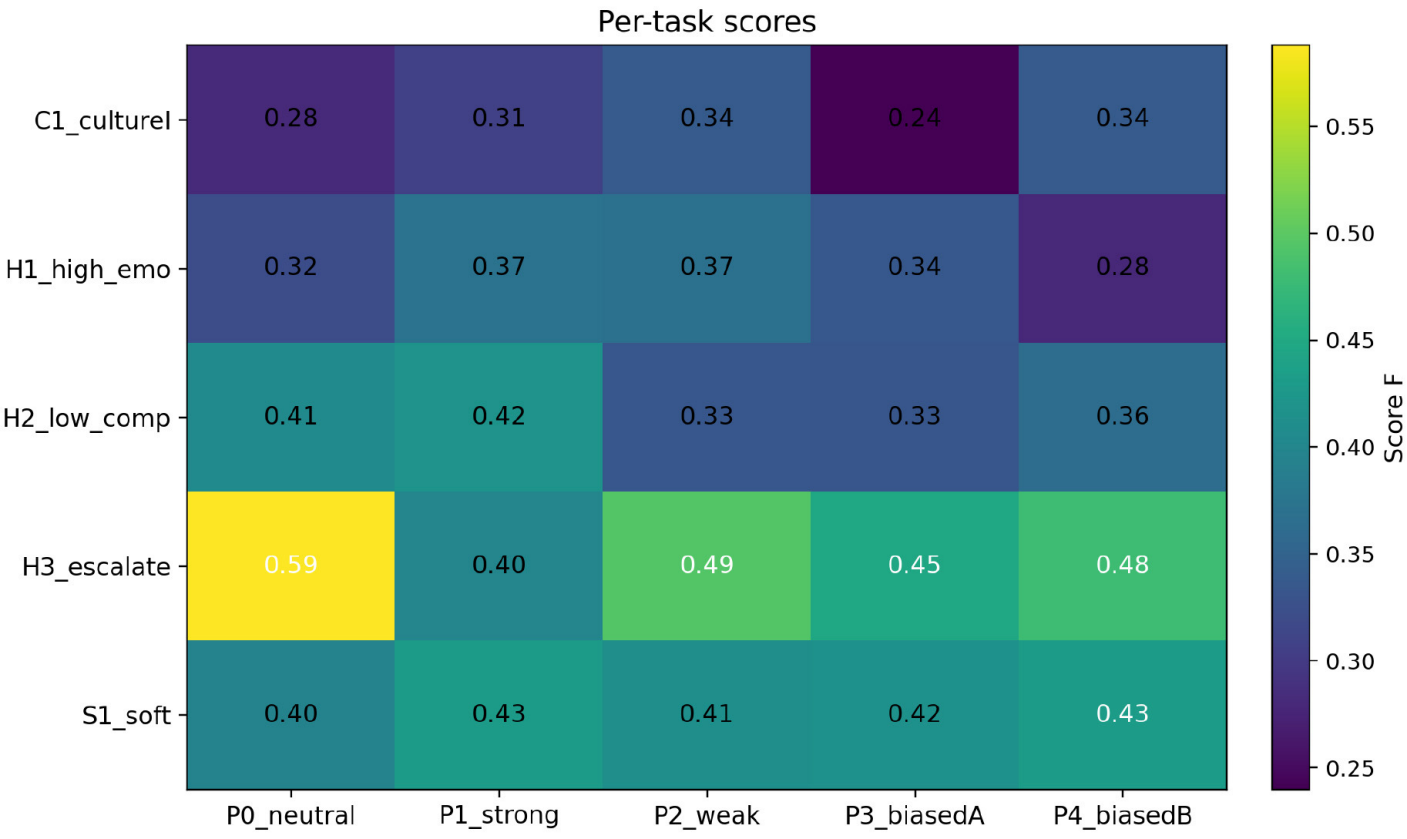
Task-wise composite scores across policies [tasks S1/H1/H2/H3/C1; *N* = 240 per cell, *T*_max_ = 40, ω = (1.0, 0.6, 0.2, 0.2)].

Per-task scores are shown in Figure 2. The neutral policy P0 performs particularly well on the escalation task H3, while P2 remains competitive across all tasks and tends to improve the speed term without incurring a large inequality penalty. By contrast, the biased policies (P3, P4) exhibit markedly larger inequality deductions across multiple tasks, thereby depressing the composite score under the fixed weights.

**Figure 3:**
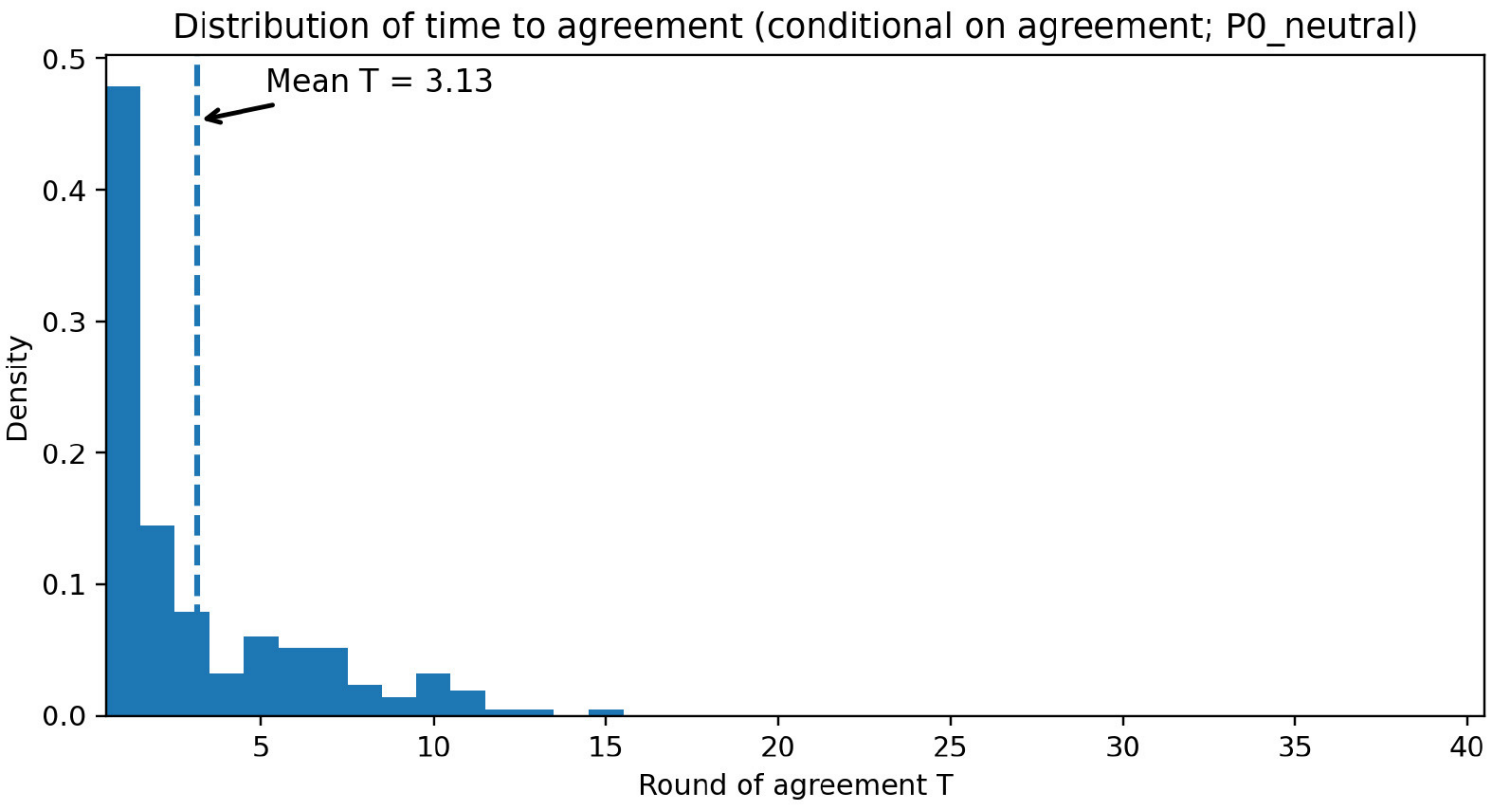
Distribution of conditional time to agreement under the neutral reference policy (P0). The vertical dashed line marks the mean round. Settings: tasks S1/H1/H2/H3/C1; *N* = 240 per cell; *T*_max_ = 40.

The conditional time-to-agreement distribution under the neutral reference (P0) is concentrated in the early rounds, with a right tail (Figure 3); the vertical dashed line marks the mean conditional round (shown in the figure). This distributional view complements the task-wise composites by making clear that speed differences arise mainly from shifting mass in early rounds rather than eliminating the right tail.

**Figure 4:**
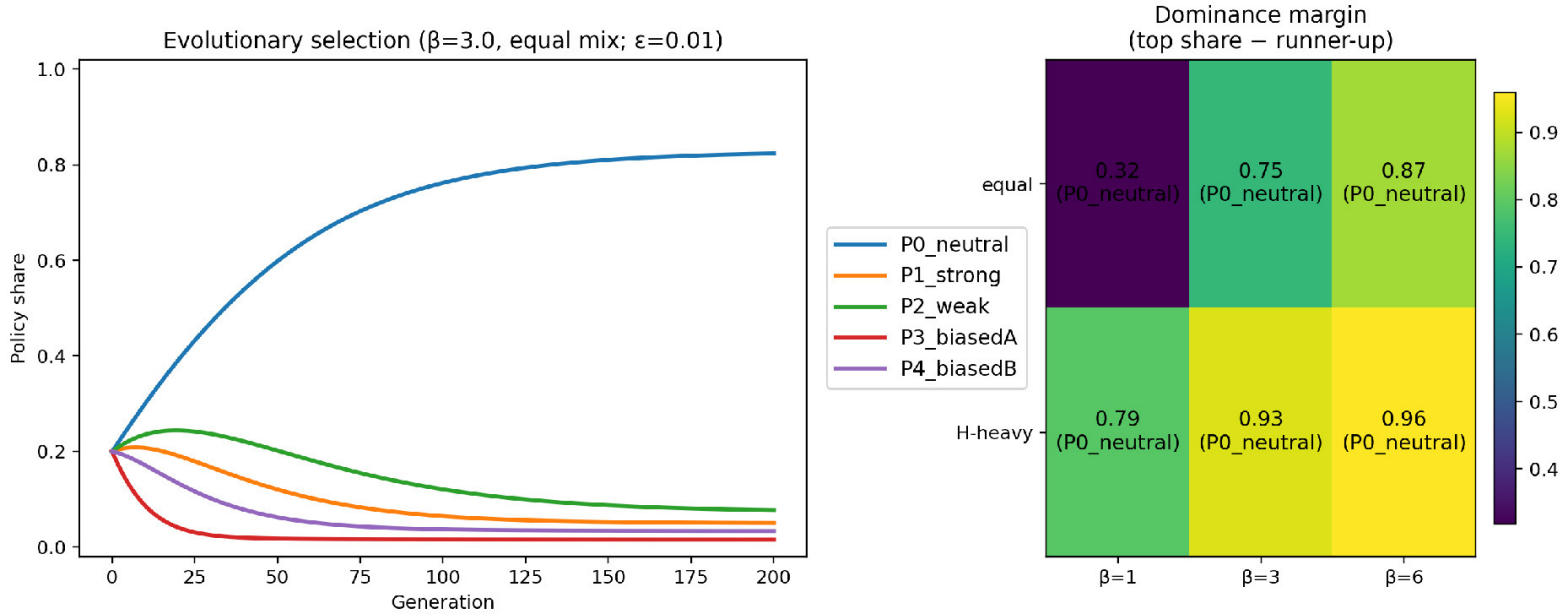
Evolutionary policy shares under the Fermi update (β = 3.0, *G* = 200) induced by the same composite score; task mix S1/H1/H2/H3/C1; *N* = 240 per cell; ω = (1.0, 0.6, 0.2, 0.2).

Under the Fermi update with selection intensity β = 3.0 for *G* = 200 generations (Figure 4), the policy shares evolve according to the same composite score used for evaluation. In this configuration, the dominant policy in the evolutionary selector coincides with the top-scoring policy in Figure 1, and the dominance pattern is illustrated here at β = 3.0.

Across figures and the summary table, the ranking is not influenced by a single outlier term but by how the same four endpoints trade off under fixed weights across tasks. Because ${\hat{F}}_{t}\left(\pi \right)$ uses identical endpoints and identical weights for every task and policy, bar heights in Figure 1 are directly comparable.

Under the equal task mix, the neutral reference P0 attains the highest task-averaged score B (Table 2), with the weak-intervention policy P2 as a close runner-up. The main contrast between P0 and P2 is a small trade-off between the first two terms of Equation 18: P2 tends to reduce conditional time, whereas P0 attains a slightly higher agreement probability. The biased policies (P3, P4) are penalized primarily through larger inequality deductions, which lowers their composite score under the fixed weights.

Task heterogeneity clarifies where these differences arise (Figure 2). P0 shows its greatest advantage on the escalation landscape H3, where its composite score exceeds that of the other policies by a wide margin. P2 remains consistently competitive across tasks and is often second-best. On the high-reactivity task H1, the best-performing policy switches: P1 attains the highest ${\hat{F}}_{\text{H}1}$ in our point estimates, but the margin over P2 is small, suggesting that weak and strong intervention behave similarly in this particular landscape at the present calibration.

The distributional view in Figure 3 complements the task-wise bars by showing that speed differences arise mainly from shifting probability mass toward early agreements rather than eliminating the right tail. This supports the interpretation that policies differ primarily in how quickly they reach the acceptance region upon agreement.

Figure 4 re-expresses the same composite score as a population dynamic under the Fermi update. Because evolutionary selection uses the same objective as evaluation, the dominant policy under the selector coincides with the top-ranked policy in Figure 1 for the equal task mix; in our configuration, P0 approaches dominance at β = 3.0 (Figure 4).

Uncertainty quantification indicates that the top two policies are close at the present sampling budget. We computed percentile bootstrap confidence intervals for the task-mix average B using 10,000 bootstrap replicates under fixed task mixes (equal and H-heavy), resampling the episode-level outcomes within each task–policy cell and aggregating them according to the specified mix. The resulting interval for Δ=B(P0)-B(P2) includes zero under both mixes (Table 3), so we report P0 as the top-ranked policy by point estimate while treating P2 as a statistically indistinguishable runner-up at *N* = 240 per cell. To support auditability under the no-supplement constraint, Appendix A enumerates the exact CSV artifacts (including episode logs), and Appendix B provides the full end-to-end reproduction script.

**Table 3:** Bootstrap confidence intervals for Δ=B(P0)--B(P2) under two task mixes.

Task mix	Mean	CI_2.5*%*_	CI_97.5*%*_
Equal mix	0.0062	–0.0519	0.0601
H-heavy mix	0.0381	–0.0283	0.1059

### 3.2 Benchmark definition

Let $T=\left\{t_{1},\ldots ,t_{m}\right\}$ be the predefined task set (S1, H1, H2, H3, C1). For task t∈T and policy π, we simulate *N* independent episodes *k* = 1, …, *N* and record: agreement $A_{t,k}^{\pi }\in \left\{0,1\right\}$, agreement round $T_{t,k}^{\pi }\in \mathbb{N}$ (if $A_{t,k}^{\pi }=1$), final settlement $y_{t,k}^{*,\pi }\in \left[0,1\right]$ (if agreed), and per–round emotions $e_{t,k,\cdot }^{A,\pi },e_{t,k,\cdot }^{B,\pi }$.

For an episode that ends at *T* ≥ 2, the emotional amplitude is


$$\begin{array}{c} Vol\left(e_{\cdot }\right)=\frac{1}{2\left(T-1\right)}\underset{i\in \left\{A,B\right\}}{\sum }\overset{T-1}{\underset{u=1}{\sum }}\left|e_{u+1}^{i}-e_{u}^{i}\right|. \end{array}$$ (14)

Task–level estimators are


$$\begin{array}{c} {\hat{\mathrm{Pr}}}_{t}\left(\pi \right)=\frac{1}{N}\overset{N}{\underset{k=1}{\sum }}A_{t,k}^{\pi }, \end{array}$$ (15)

$$\begin{array}{c} {\hat{𝔼}}_{t}\left[T|\text{Agree},\pi \right]=\frac{\sum_{k=1}^{N}A_{t,k}^{\pi }T_{t,k}^{\pi }}{\sum_{k=1}^{N}A_{t,k}^{\pi }}, \end{array}$$ (16)

$$\begin{array}{c} {\hat{\text{Ineq}}}_{t}\left(\pi \right)=\frac{\sum_{k=1}^{N}A_{t,k}^{\pi }\left|y_{t,k}^{*,\pi }-\frac{1}{2}\right|}{\sum_{k=1}^{N}A_{t,k}^{\pi }}, \end{array}$$ (17)

$$\begin{array}{c} {\hat{\text{Vol}}}_{t}\left(\pi \right)=\frac{\sum_{k=1}^{N}A_{t,k}^{\pi }\text{Vol}\left(e_{\cdot }\right)}{\sum_{k=1}^{N}A_{t,k}^{\pi }}. \end{array}$$ (18)

No–agreement case convention: if $\underset{k}{\sum }A_{t,k}^{\pi }=0$, then set $({\hat{𝔼}}_{t}\left[T|\text{Agree},\pi \right]{)}^{-1}={\hat{\text{Ineq}}}_{t}\left(\pi \right)={\hat{\text{Vol}}}_{t}\left(\pi \right)=0$.

Given non-negative weights ω = (ω_1_, ω_2_, ω_3_, ω_4_), the task–level composite score is


$$\begin{array}{l} {\hat{F}}_{t}\left(\pi \right)=\omega_{1}{\hat{\mathrm{Pr}}}_{t}\left(\pi \right)+\omega_{2}({\hat{𝔼}}_{t}\left[T|\text{Agree},\pi \right]{)}^{-1}\\ \;-\omega_{3}{\hat{\text{Ineq}}}_{t}\left(\pi \right)-\omega_{4}{\hat{\text{Vol}}}_{t}\left(\pi \right). \end{array}$$ (19)

The overall benchmark score, cross–task robustness, and worst–task lower bound are


$$\begin{array}{c} B\left(\pi \right)=\frac{1}{\left|T\right|}\underset{t\in T}{\sum }{\hat{F}}_{t}\left(\pi \right), \end{array}$$ (20)

$$\begin{array}{c} {\hat{\text{CV}}}_{\mathrm{Pr}}\left(\pi \right)=\frac{\text{s}{\text{d}}_{t}({\hat{\mathrm{Pr}}}_{t}\left(\pi \right))}{\text{mea}{\text{n}}_{t}({\hat{\mathrm{Pr}}}_{t}\left(\pi \right))+\varepsilon },\;\hat{\text{Robust}}\left(\pi \right)=\exp(-{\hat{\text{CV}}}_{\mathrm{Pr}}\left(\pi \right)), \end{array}$$ (21)

$$\begin{array}{c} W\left(\pi \right)=\underset{t\in T}{\min}{\hat{F}}_{t}\left(\pi \right), \end{array}$$ (22)

with a small ε > 0 for numerical stability. In our runs we fix *N* = 240, *T*_max_ = 40, and ω = (1.0, 0.6, 0.2, 0.2); results are summarized in Figures 1–4 and Table 2.

The endpoints and weights are chosen to price process control directly, rather than only the quality of the candidate point. Agreement probability rewards reaching settlements; the speed term rewards doing so without consuming excessive rounds; the inequality deduction prices distributional skew; the volatility deduction prices emotional turbulence. Because all four are computed identically for every policy and task, the benchmark is auditable and resists *ad hoc post-hoc* criteria. This is the sense in which the evaluation goes “beyond mediation”: it internalizes side-effects on fairness and effects that are typically external to single-metric evaluations.

Evolutionary selection uses exactly the same endpoints. Under the discrete Fermi rule, any consistent edge in the composite score increases a policy's share multiplicatively across generations. A policy that shortens time but systematically worsens inequality would see its per-session advantage offset in the composite and would not diffuse widely. Figure 4 does not introduce a new objective; it maps the same multi-criterion score into long-run adoption under social learning.

The benchmark is designed to be portable and auditable. Tasks T represent generic “hard spots” that are commonly encountered and mechanistically distinct (reactivity, fatigue, escalation, cultural asymmetry). Each endpoint is defined by a simple statistic that can be recomputed from raw episode logs, and the aggregation Equations 18, 19 is fixed ex ante. As a result, reproducing the ranking amounts to rerunning the Monte Carlo with publicly specified seeds, initial conditions, and parameter files; no manual tuning or researcher degrees of freedom are required after the configuration is frozen.

Beyond single-session evaluation, the same composite feeds an evolutionary selector via Equation 11. This mapping is intentional: if a policy achieves its apparent success by shifting mass toward faster but skewed settlements, the inequality deduction reduces its *F* and therefore its adoption probability under the Fermi rule. Conversely, policies whose small advantages are steady across landscapes (as for P2) benefit from compounding. Figure 4 should therefore be read not as a separate model but as the long-run implication of the same multi-criterion score under social learning.

Two diagnostic quantities help interpret the aggregate numbers. The cross-task coefficient of variation in Equation 20 captures whether a policy's agreement probability is brittle, highly successful on some tasks but failing elsewhere, or steady across the case mix. The worst-task lower bound Equation 21 answers the decision-maker's question, “How bad can this policy be on a difficult day?” Reporting B(π), $\hat{\text{Robust}}\left(\pi \right)$, and W(π) side by side thus separates average performance, stability, and downside risk.

While the present release sets ω = (1.0, 0.6, 0.2, 0.2) to balance agreement, speed, fairness, and affect, the code accepts user-specified weights to reflect different institutional priorities (e.g., stricter fairness constraints). Because the decomposition is linear, changing ω preserves interpretability: contributions from each endpoint can be audited and traced to the underlying logs. This property is essential for deployment choices that must be explained to practitioners and oversight bodies.

External validity is addressed by the way the benchmark separates mechanisms. Reactivity (H1), fatigue (H2), escalation (H3), and cultural asymmetry (C1) are not tuned to produce a particular winner; they are generic stressors shared by many mediation settings. Institutions that prioritize different outcomes can change the weights ω without altering the endpoint definitions or the logging interface, preserving comparability while reflecting local norms (e.g., stricter fairness penalties). Because the mapping from raw logs to ${\hat{F}}_{t}\left(\pi \right)$ is linear and transparent, any alternative weighting can be audited ex post by recomputing contributions from the released episode-level files. This portability is central to the “beyond mediation” goal: the same protocol can be rerun with different priors and yet remain falsifiable, since deviations in ranking can be traced to explicit choices about what to value (speed, fairness, or affect) rather than undocumented implementation details.

### 3.3 Interpretation of results

Under the equal task mix and fixed weights ω = (1.0, 0.6, 0.2, 0.2), the neutral reference policy P0 (*b* = 0, γ = 0.6) attains the highest task-averaged composite score B (Table 2 and Figure 1). The weak-intervention policy P2 (*b* = 0, γ = 0.2) is a close runner-up. In Table 2, P0 achieves B=0.399 with Pr(Agree) = 0.179 and 𝔼[*T* ∣ Agree] = 2.90, whereas P2 achieves B=0.390 with Pr(Agree) = 0.174 and 𝔼[*T* ∣ Agree] = 2.66; the 95% bootstrap confidence intervals for B overlap substantially (Table 2), confirming that the top two policies are statistically close at the present sampling budget. This pattern makes the benchmark trade-off explicit: P0 is slightly better on agreement probability, while P2 is slightly faster conditional on agreement, and the net effect favors P0 under the fixed weights.

The strong-intervention policy P1 (*b* = 0, γ = 0.9) illustrates why “pushing harder” does not automatically maximize the composite. Although P1 attains the highest task-averaged agreement probability in Table 2 (Pr(Agree) = 0.192), it incurs larger deductions through inequality and volatility (Ineq = 0.006, Vol = 0.138), yielding a lower B=0.386 than P0 and P2. The biased policies P3 and P4 are penalized primarily through substantially larger inequality (Table 2), which depresses their composite scores despite gains in other components, leaving them below the bias-free policies in the overall ordering.

Task heterogeneity clarifies where the margins arise (Figure 2). P0 shows its largest advantage on the escalation landscape H3, while P2 remains consistently competitive across tasks and often improves the speed component without incurring a large inequality penalty. On the high-reactivity task H1, the best-performing policy switches by point estimate: P1 attains the highest ${\hat{F}}_{\text{H}1}$ in our calibration, but the margin over P2 is small, indicating that weak and strong interventions behave similarly on that particular landscape under the current parameters.

The conditional time-to-agreement distribution (Figure 3) indicates that policy differences arise mainly from shifting probability mass toward earlier agreements rather than eliminating the right tail entirely. Conversely, intervention strength primarily affects how quickly trajectories enter the acceptance region upon agreement, not whether hard cases disappear.

From a robustness perspective, the indicator derived from the across-task variation in agreement probability, exp(−CV), is highest for the strong-intervention policy, consistent with the interpretation that P1 avoids large collapses across landscapes even though it is not top-ranked by B. Taken together, the task-wise bars and the summary table support a practical reading: default to a bias-free style (P0/P2), treat stronger interventions as context-dependent rather than universally optimal, and avoid bias, as inequality penalties dominate under the present aggregation.

In the present calibration, the volatility term contributes relatively little to the ranking because damping and progress-based calming keep Vol in a narrow range (Table 2). If one wishes to discriminate emotional stability more strongly, one would need to weaken calming coefficients, increase noise variance, or add task conditions that activate larger affect fluctuations; doing so would increase the leverage of the volatility deduction relative to the other components, without changing the benchmark definition.

Overall, benchmark v1 functions as an instrument for comparing AI-mediator operating policies by fixing (i) a predefined task set, (ii) auditable endpoints, and (iii) a pre-specified linear aggregation. The close gap between P0 and P2 under the current sampling budget (Table 3) suggests that the top two policies are statistically close at *N* = 240 per cell, and therefore deployment choices should be guided by the expected case mix rather than by point estimates alone.

## 4 Discussion

Under the equal task mix (S1/H1/H2/H3/C1) and fixed weights ω = (1.0, 0.6, 0.2, 0.2), the neutral reference policy (P0; *b* = 0, γ = 0.6) attains the highest task-averaged score B (Figure 1 and Table 2). The weak-intervention policy (P2; *b* = 0, γ = 0.2) is a close runner-up, reflecting a small trade-off between agreement probability and conditional speed: P0 is slightly higher on Pr(Agree), whereas P2 is slightly faster in 𝔼[*T* ∣ Agree] (Table 2). The strong-intervention policy (P1; *b* = 0, γ = 0.9) achieves the highest task-averaged agreement probability, but its overall score remains below P0/P2 because larger deductions due to inequality and volatility accumulate under the fixed aggregation. The biased policies (P3, P4) are ranked lower primarily because inequality penalties dominate their gains in other components (Table 2). All deployment-oriented statements in this section are conditional on the pre-registered task mix and weight vector ω, and should be read as decision support rather than normative prescriptions.

Task heterogeneity clarifies where these margins arise (Figure 2). P0 shows its greatest advantage on the escalation landscape H3, while P2 remains consistently competitive across tasks by improving the speed component without incurring a large inequality penalty. On the high-reactivity task H1, the best-performing policy switches by point estimate: P1 attains the highest ${\hat{F}}_{\text{H}1}$, but the margin over P2 is small, indicating that weak and strong interventions behave similarly on that landscape under the current calibration.

The benchmark is important because it fixes both the comparison set and the aggregation rule. Policies are evaluated on the same predefined task set, and the composite Equation 18 prices four endpoints: agreement frequency, conditional speed 𝔼(*T* ∣ Agree)^−1^, distributional equity, and emotional turbulence. Averaging Equation 18 across tasks yields B(π) in Equation 19, while the robustness statistic in Equation 20 complements the mean by quantifying steadiness across case mix. This makes value choices explicit through ω and separates average performance from stability.

A separate question is stability across the case mix. The robustness summary in Equation 20 complements the mean score by capturing cross-task variability in agreement probability, while the worst-task bound W(π) in Equation 21 makes downside risk explicit. In Table 2, the strong intervention shows the highest robustness index while retaining a lower mean score, whereas the bias-free baselines combine higher mean performance with acceptable robustness. Therefore, the choice of deployment depends on the environment: when the case mix is broad and downside risk is costly, stability may be more important; when context-adaptive operation is feasible, selecting the mean performer is more justified.

Several mechanisms explain the ordering. In this v1 model, we used “trust repair” as an operational shorthand for the recovery of a cooperative state after conflict, proxied by willingness recovery and affective calming that restore acceptance propensity in subsequent rounds (Equations 6, 5). Because *D*_*t*_ and *e*_*i,t*_ enter utility with the same sign as distance (Equation 3), interventions that shorten time by raising tension or arousal can pay back through lower acceptance or more skewed settlements. Willingness *c*_*i,t*_ recovers only when the candidate is perceived as an improvement (Equation 6), and the progress flag Γ_*t*_ provides a calming pathway that helps prevent escalation in *D*_*t*_ (Equation 7). These channels imply that stronger control is not monotone in overall performance.

The H1 inversion provides valuable insights. When the reactivity parameters ψ_*i*_ are large, even moderate proposal gaps raise arousal quickly, and the logistic acceptance in Equation 4 becomes highly sensitive to transient utility movements. In this landscape, stronger intervention (P1; γ = 0.9) attains the highest ${\hat{F}}_{\text{H}1}$ by point estimate (Figure 2). A plausible mechanism is that a larger anchor-to-midpoint shrink moves trajectories into the acceptance region earlier when agreement occurs, partially offsetting the escalation of *e*_*i,t*_ and *D*_*t*_. The benchmark also shows why this does not translate into the best task-average performance: across the full task set, the same strong control accumulates larger deductions due to inequality and volatility (Table 2), so its net composite remains below the bias-free baselines under the fixed weights.

The evolutionary plot (Figure 4) connects session-level performance to long-run adoption under the Fermi update (Equation 11) using the same payoff *F*(π). Because Equation 18 adds agreement and speed while subtracting inequality and volatility, small but consistent net advantages compound over generations. This links process variables (*e*_*i,t*_, *c*_*i,t*_, *D*_*t*_) to population-level consequences without introducing a new objective.

To support interpretability and empirical anchoring, we map model primitives to observables. Proposals (*x*_*i,t*_, *s*_*t*_) and agreement timing are directly logged, inequality is literal for numeric splits (or uses a documented embedding to [0, 1]), and emotional amplitude uses total variation of coded affect (Equation 13). With these logs, Table 2 can be recomputed via Equations 14–17 and aggregated by Equations 18, 19.

In future empirical validation, we will log offer trajectories (*x*_*i,t*_, *s*_*t*_), acceptance timing, and lightweight affect codes (or sentiment proxies) in mediated sessions, and estimate key task parameters by matching the joint distribution of $\left(A,T_{\text{agree}},y^{*}\right)$ and volatility statistics. This enables falsification of the predefined task landscapes (reactivity, fatigue, escalation, asymmetry) rather than *post-hoc* fitting. We will report performance under a small pre-registered grid of ω values aligned with stakeholder priorities.

### 4.1 Descriptive benchmark findings vs. normative deployment choices

These results are descriptive for the benchmark fixed in Sections 2 and 3: they summarize trade-offs under a controlled simulation and a declared weight vector ω. Any deployment guidance is therefore conditional on governance priorities (speed vs. fairness, mean performance vs. downside risk). A cautious reading is to default to a bias-free neutral/weak control and treat stronger interventions or slight framing as context-dependent options whose fairness cost should be monitored. Reporting B(π) together with $\hat{\text{Robust}}\left(\pi \right)$ and W(π) makes these trade-offs explicit.

Conditional guidance can nevertheless be stated carefully. These results suggest a plausible operating heuristic under the benchmark's assumptions: neutral/weak intervention is a reasonable default, while stronger control or slight framing may be useful in specific regimes (e.g., high reactivity), provided that the implied fairness trade-off is monitored. We emphasized that such guidance is conditional on the pre-specified task mix and the aggregation weights, and should be treated as decision support rather than a universal prescription. Reporting B(π) together with a cross-task robustness statistic like Equation 20 and the worst–task bound W(π) in Equation 21 helps stakeholders see average performance, steadiness across case mix, and downside protection on the same page. For data collection, pre-register the task set and weights ω to avoid *post-hoc* tailoring, keep the Monte Carlo sample size large enough that the *O*(*N*^−1/2^) concentration observed in the simulations holds in finite samples, and archive seeds and configuration files so that figures analogous to Figures 1–4 can be regenerated.

There are certain limitations in this study, which suggest immediate extensions. The one-dimensional proposal simplifies multi-issue disputes; nonetheless, it is sufficient to surface the core trade-off between speed and fairness mediated by *c*_*i,t*_ and *e*_*i,t*_. The logistic acceptance abstracts comprehension and legal/financial constraints. This is standard in discrete choice and can be refined by adding a shared shock to model correlated acceptance when both parties respond to the same momentary event. In the present calibration, the contribution of emotional amplitude Vol is small because damping is relatively strong and noise is modest; this is expected under the current settings. The benchmark can be made more sensitive to emotional turbulence through pre-registered adjustments to these parameters or by introducing event-like perturbations (e.g., caucus shocks or third-party information) that stress the dynamics. Finally, the benchmark's weights reflect one set of priorities; sensitivity analysis indicates that the qualitative ranking is stable under moderate perturbations, but a field deployment should report results across a small grid of ω values to document value dependence explicitly.

In summary, the benchmark offers a reproducible, multi-criterion method for comparing mediation styles that extends beyond the candidate settlement itself, incorporating the dynamics of emotion, willingness, and tension. The weak-intervention style emerges as the best-performing average across representative hard cases and remains competitive under evolutionary adoption, while targeted, reversible framing plays a role in high-reactivity regimes. These findings are actionable, falsifiable, and can be expanded to more complex settings where multi-issue structures, event shocks, or institutional constraints are integrated into the same measurement and aggregation framework.

## Data Availability

The raw data supporting the conclusions of this article will be made available by the authors, without undue reservation.
