# Supplementary material for: Beyond mediation: an evolutionary benchmark for emotionally and normatively competent AI
Source: Front Artif Intell. 2026 Apr 23;9:1719225. doi: 10.3389/frai.2026.1719225 (PMC13149239; doi:10.3389/frai.2026.1719225)
Supplement: Supplementary file 1 [file Data_Sheet_1.pdf]

APPENDIX A. REPRODUCIBILITY ARTIFACTS (CSV OUTPUTS)

This manuscript is subject to a no-supplement constraint; therefore, we enumerate the exact file artifacts required to regenerate all reported tables and figures.

Deterministic configuration

- `run_config.json`: full run configuration (N, seed, bootstrap replicates, weights, evolutionary settings).

Task-wise aggregates (used by Tables/Figs in the main text)

- `per_task_metrics.csv`: task-level estimates for each policy, including $\widehat{\Pr}_t(\pi)$, $\widehat{\mathbb{E}}[T \mid \text{Agree}, \pi]$, $\widehat{\text{Ineq}}_t(\pi)$, $\widehat{\text{Vol}}_t(\pi)$, and $\widehat{F}_t(\pi)$ for $t \in \{\text{S1}, \text{H1}, \text{H2}, \text{H3}, \text{C1}\}$.
- `scoreboard_equal.csv`: policy ranking and aggregation under the equal task mix; used to produce Table 2 and Fig. 1.
- `scoreboard_Hheavy.csv`: policy ranking and aggregation under the H-heavy task mix; used for robustness checks reported in the text.

Bootstrap outputs (percentile confidence intervals)

- `bootstrap_overallF_equal.csv`: bootstrap replicates of $\mathscr{B}(\pi)$ under the equal mix (percentile CIs).
- `bootstrap_overallF_Hheavy.csv`: bootstrap replicates of $\mathscr{B}(\pi)$ under the H-heavy mix (percentile CIs).
- `bootstrap_diff_P0minusP2_equal.csv`: bootstrap replicates of $\Delta = \mathscr{B}(\text{P0}) - \mathscr{B}(\text{P2})$ under the equal mix; summarized in Table 3.
- `bootstrap_diff_P0minusP2_Hheavy.csv`: bootstrap replicates of Δ under the H-heavy mix; summarized in Table 3.

Episode-level logs (required for Fig. 3 and bootstrap auditability)

- Directory `episodes/` containing `episodes_{policy}__{task}.csv` for every task–policy cell. Each row stores `A` (agreement indicator), `T_agree` (round of agreement; set to $T_{\max}$ if no agreement), `Ineq` and `Vol` (defined on agreeing episodes; `NaN` otherwise).

B END-TO-END REPRODUCTION SCRIPT

The following listing provides the full end-to-end reproduction script. Dependencies: Python 3.x with `numpy`, `pandas`, and `matplotlib`. Random seeds are fixed for exact regeneration.

```
# -*- coding: utf-8 -*-
# REVIEW-READY FULL SCRIPT (COLAB/JUPYTER OK) -- Fig4 legend centered
# - Runs all taskxpolicy cells
# - Saves per-episode CSVs (for bootstrap + Fig3)
# - Outputs CSVs + 4 figures with fixes:
#   FigA: overall bar (labels not cut off)
#   FigB: heatmap with per-cell numbers (auto text color)
#   Fig3: T histogram with mean value label/arrow
#   Fig4: evolution + dominance; LEGEND CENTERED inside LEFT panel (axL)
#
# deps: numpy, pandas, matplotlib
#
# Colab usage:
#   !python review_run.py --out ./out_review --N 240 --seed 123 --B 2000
# or in a notebook cell:
#   main()   (it ignores kernel -f args via parse_known_args())

import os, argparse, json, hashlib
import numpy as np
import pandas as pd
import matplotlib.pyplot as plt
from dataclasses import dataclass, asdict

plt.rcParams["axes.unicode_minus"] = False

# ========= utils =========
def logistic(z: float) -> float:
    return 1.0 / (1.0 + np.exp(-z))

def _stable_int_seed(*parts, mod=2**32):
    s = "|".join(str(p) for p in parts).encode("utf-8")
    h = hashlib.blake2b(s, digest_size=8).digest()
    return int.from_bytes(h, "little") % mod

def ci_percentile(x, lo=2.5, hi=97.5):
    return float(np.percentile(x, lo)), float(np.percentile(x, hi))

# ========= model =========
@dataclass
class Params:
    # Behavioral / psychological
    kA: float = 4.0; kB: float = 4.0
    tauA: float = -0.35; tauB: float = -0.35
    lamA: float = 0.2; lamB: float = 0.2
    muA: float = 0.3; muB: float = 0.3
    rhoA: float = 0.8; rhoB: float = 0.8
    psiA: float = 0.6; psiB: float = 0.6
    chiA: float = 0.3; chiB: float = 0.3
    deltaA: float = 0.05; deltaB: float = 0.05
    alphaA: float = 0.6; alphaB: float = 0.6
    betaA: float = 0.8; betaB: float = 0.8

    # Mediator
    b: float = 0.0
    gamma: float = 0.6

    # Noise
    sigA: float = 0.05; sigB: float = 0.05
    nuA: float = 0.01; nuB: float = 0.01

    # Conflict
    eta0: float = 0.3; eta1: float = 0.6
```

```
    # Progress threshold (joint utility improvement)
    eps_prog: float = 0.0

    # Initial states
    xA0: float = 0.15; xB0: float = 0.85
    eA0: float = 0.2;  eB0: float = 0.2
    cA0: float = 0.5;  cB0: float = 0.5
    D0: float = 0.0

    # Horizon
    T_max: int = 40

def mediator_point(xA: float, xB: float, b: float, gamma: float) -> float:
    # paper-like convex blend:
    # w=(1+b)/2, m=w*xA+(1-w)*xB, xbar=(xA+xB)/2, s=(1-gamma)*m + gamma*xbar
    w = (1.0 + b) / 2.0
    m = w * xA + (1.0 - w) * xB
    xbar = 0.5 * (xA + xB)
    s = (1.0 - gamma) * m + gamma * xbar
    return float(np.clip(s, 0.0, 1.0))

def episode(P: Params, rng: np.random.Generator):
    xA, xB = P.xA0, P.xB0
    eA, eB = P.eA0, P.eB0
    cA, cB = P.cA0, P.cB0
    D = P.D0

    prev_eA, prev_eB = eA, eB
    vol_sum = 0.0
    n_steps = 1
    prev_jointU = None

    for t in range(1, P.T_max + 1):
        s = mediator_point(xA, xB, P.b, P.gamma)

        UA = -abs(s - 0.0) - P.lamA * D - P.muA * eA
        UB = -abs(s - 1.0) - P.lamB * D - P.muB * eB

        pA = logistic(P.kA * (UA - P.tauA))
        pB = logistic(P.kB * (UB - P.tauB))

        agree = (rng.random() < pA) and (rng.random() < pB)
        if agree:
            vol = 0.0 if n_steps <= 1 else vol_sum / (2.0 * (n_steps - 1))
            return dict(A=1, T_agree=t, Ineq=abs(s - 0.5), Vol=vol)

        jointU = float(UA + UB)
        if prev_jointU is None:
            progress = 0.0
        else:
            progress = 1.0 if (jointU - prev_jointU) >= P.eps_prog else 0.0
        prev_jointU = jointU

        Delta = xA - xB
        eA = P.rhoA * eA + P.psiA * abs(Delta) - P.chiA * progress + rng.normal(0.0, P.sigA)
        eB = P.rhoB * eB + P.psiB * abs(Delta) - P.chiB * progress + rng.normal(0.0, P.sigB)
        eA = max(float(eA), 0.0)
        eB = max(float(eB), 0.0)

        vol_sum += abs(eA - prev_eA) + abs(eB - prev_eB)
        prev_eA, prev_eB = eA, eB
        n_steps += 1

        PhiA = abs(s - 0.0) - abs(xA - 0.0)
        PhiB = abs(s - 1.0) - abs(xB - 1.0)
        cA = (1.0 - P.deltaA) * cA + P.alphaA * PhiA
        cB = (1.0 - P.deltaB) * cB + P.alphaB * PhiB
```

```
        cA = float(np.clip(cA, 0.0, 1.0))
        cB = float(np.clip(cB, 0.0, 1.0))

        D = max(D + P.eta0 * (1.0 - pA * pB) - P.eta1 * (pA * pB), 0.0)

        xA = float(np.clip(xA + P.betaA * cA * (s - xA) + rng.normal(0.0, P.nuA), 0.0, 1.0))
        xB = float(np.clip(xB + P.betaB * cB * (s - xB) + rng.normal(0.0, P.nuB), 0.0, 1.0))

    return dict(A=0, T_agree=P.T_max, Ineq=np.nan, Vol=np.nan)

def run_cell_episode_df(P: Params, N: int, seed: int) -> pd.DataFrame:
    rng = np.random.default_rng(seed)
    rows = [episode(P, rng) for _ in range(N)]
    return pd.DataFrame(rows)

def metrics_from_episode_df(ep: pd.DataFrame) -> dict:
    N = len(ep)
    N_agree = int((ep["A"] == 1).sum())
    Pr = N_agree / N
    if N_agree > 0:
        ET = float(ep.loc[ep["A"] == 1, "T_agree"].mean())
        Ineq = float(ep.loc[ep["A"] == 1, "Ineq"].mean())
        Vol = float(ep.loc[ep["A"] == 1, "Vol"].mean())
    else:
        ET = np.nan; Ineq = np.nan; Vol = np.nan
    return dict(Pr=Pr, ET=ET, Ineq=Ineq, Vol=Vol)

def score(m: dict, w):
    ET = m.get("ET", np.nan)
    invT = 0.0 if (np.isnan(ET) or ET <= 0.0) else 1.0 / ET
    Ineq = 0.0 if np.isnan(m.get("Ineq", np.nan)) else float(m["Ineq"])
    Vol  = 0.0 if np.isnan(m.get("Vol",  np.nan)) else float(m["Vol"])
    return float(w[0]*m["Pr"] + w[1]*invT - w[2]*Ineq - w[3]*Vol)

# ========= tasks & policies =========
BASE = Params()
TASKS = {
    "S1_soft":     Params(**{**asdict(BASE), "psiA":0.5, "psiB":0.5, "eta0":0.25, "eta1":0.7}),
    "H1_high_emo": Params(**{**asdict(BASE), "psiA":1.1, "psiB":1.1}),
    "H2_low_comp": Params(**{**asdict(BASE), "alphaA":0.3, "alphaB":0.3, "deltaA":0.12, "deltaB":0.12}),
    "H3_escalate": Params(**{**asdict(BASE), "eta0":0.6, "eta1":0.3}),
    "C1_cultureI": Params(**{**asdict(BASE), "tauA":-0.50, "tauB":-0.10, "alphaA":0.5, "alphaB":0.7, "psiA":0.55, "
}
POLICIES = {
    "P0_neutral": {"b":0.0,  "gamma":0.6},
    "P1_strong":  {"b":0.0,  "gamma":0.9},
    "P2_weak":    {"b":0.0,  "gamma":0.2},
    "P3_biasedA": {"b":0.2,  "gamma":0.6},
    "P4_biasedB": {"b":-0.2, "gamma":0.6},
}

def task_mix_equal(tasks):
    return {t: 1.0/len(tasks) for t in tasks}

def task_mix_Hheavy(tasks):
    H = [t for t in tasks if t.startswith("H")]
    w = {t: 0.0 for t in tasks}
    if not H:
        return task_mix_equal(tasks)
    for t in H:
        w[t] = 1.0/len(H)
    return w

def policy_overall_from_cellF(df_cellF: pd.DataFrame, mix: dict) -> pd.Series:
    piv = df_cellF.pivot(index="task", columns="policy", values="F")
    tasks = list(piv.index)
    pols = list(piv.columns)
    w = np.array([mix.get(t, 0.0) for t in tasks], dtype=float)
```

```
    w = w / w.sum()
    Fpol = (w[:, None] * piv.values).sum(axis=0)
    return pd.Series(Fpol, index=pols)

# ========= bootstrap =========
def bootstrap_overallF_from_files(episodes_dir, pols, tasks, w, mix, B, seed):
    rng = np.random.default_rng(seed)
    K = len(pols)
    overallF_boot = np.zeros((B, K), dtype=float)

    ep_store = {}
    for pol in pols:
        for task in tasks:
            path = os.path.join(episodes_dir, f"episodes_{pol}__{task}.csv")
            ep_store[(pol, task)] = pd.read_csv(path)

    for b in range(B):
        rows = []
        for pol in pols:
            for task in tasks:
                ep = ep_store[(pol, task)]
                N = len(ep)
                idx = rng.integers(0, N, size=N)
                ep_b = ep.iloc[idx].reset_index(drop=True)
                met = metrics_from_episode_df(ep_b)
                F = score(met, w)
                rows.append((pol, task, F))
        df_cellF = pd.DataFrame(rows, columns=["policy", "task", "F"])
        Fpol = policy_overall_from_cellF(df_cellF, mix)
        overallF_boot[b, :] = Fpol.loc[pols].to_numpy()

    diff_boot = None
    if ("P0_neutral" in pols) and ("P2_weak" in pols):
        i0 = pols.index("P0_neutral")
        i2 = pols.index("P2_weak")
        diff_boot = overallF_boot[:, i0] - overallF_boot[:, i2]
    return overallF_boot, diff_boot

# ========= FIGURES (fixed visuals) =========
def figA_overall_bar(df_cellF, mix, outdir, fname="figA_overall_score.png"):
    piv = df_cellF.pivot(index="task", columns="policy", values="F")
    tasks = list(piv.index)
    pols = list(piv.columns)

    w = np.array([mix.get(t, 0.0) for t in tasks], dtype=float)
    w = w / w.sum()
    overall = (w[:, None] * piv.values).sum(axis=0)

    order = np.argsort(-overall)
    pols_ord = [pols[i] for i in order]
    overall_ord = overall[order]

    fig = plt.figure(figsize=(8.2, 4.4))
    ax = fig.add_subplot(111)
    x = np.arange(len(pols_ord))
    ax.bar(x, overall_ord)

    ymax = float(np.max(overall_ord))
    for i, v in enumerate(overall_ord):
        ax.text(i, v + 0.01*ymax, f"{v:.3f}", ha="center", va="bottom", fontsize=10)

    ax.set_xticks(x)
    ax.set_xticklabels(pols_ord, rotation=0)
    ax.set_ylabel("Overall F (task-weighted)")
    ax.set_title("Benchmark overall score")
    ax.set_ylim(0.0, ymax * 1.12)

    fig.tight_layout()
```

```
    path = os.path.join(outdir, fname)
    fig.savefig(path, dpi=220)
    plt.close(fig)
    return path

def figB_task_policy_heatmap(df_cellF, outdir, fname="figB_per_task_heatmap.png",
                             fmt="{:.2f}", fontsize=11):
    pivot = df_cellF.pivot(index="task", columns="policy", values="F")

    fig = plt.figure(figsize=(9.0, 5.4))
    ax = fig.add_subplot(111)
    im = ax.imshow(pivot.values, aspect="auto", origin="upper")

    ax.set_yticks(np.arange(len(pivot.index)))
    ax.set_yticklabels(pivot.index.tolist(), fontsize=fontsize)
    ax.set_xticks(np.arange(len(pivot.columns)))
    ax.set_xticklabels(pivot.columns.tolist(), rotation=0, fontsize=fontsize)

    cbar = fig.colorbar(im, ax=ax, fraction=0.046, pad=0.04)
    cbar.set_label("Score F", fontsize=fontsize)

    ax.set_title("Per-task scores", fontsize=fontsize+2)

    vmin = np.nanmin(pivot.values)
    vmax = np.nanmax(pivot.values)
    thresh = vmin + 0.55 * (vmax - vmin)

    for i in range(pivot.shape[0]):
        for j in range(pivot.shape[1]):
            v = float(pivot.values[i, j])
            color = "white" if v >= thresh else "black"
            ax.text(j, i, fmt.format(v), ha="center", va="center",
                    fontsize=fontsize, color=color)

    fig.tight_layout()
    path = os.path.join(outdir, fname)
    fig.savefig(path, dpi=220)
    plt.close(fig)
    return path

def fig3_T_histogram_aggregate(episodes_dir, policy, tasks, T_max, outdir,
                              fname="fig3_T_distribution.png"):
    Ts_all = []
    for task in tasks:
        path = os.path.join(episodes_dir, f"episodes_{policy}__{task}.csv")
        ep = pd.read_csv(path)
        Ts = ep.loc[ep["A"] == 1, "T_agree"].to_numpy()
        if Ts.size:
            Ts_all.append(Ts)

    if not Ts_all:
        return None

    Ts_all = np.concatenate(Ts_all)
    m = float(Ts_all.mean())

    fig = plt.figure(figsize=(7.6, 4.2))
    ax = fig.add_subplot(111)

    bins = np.arange(1, T_max + 2) - 0.5
    ax.hist(Ts_all, bins=bins, density=True)

    ax.axvline(m, linestyle="--", linewidth=2.0)
    y_top = ax.get_ylim()[1]
    ax.annotate(
        f"Mean T = {m:.2f}",
        xy=(m, y_top * 0.90),
        xytext=(min(m + 2.0, T_max - 2.0), y_top * 0.98),
```

```
        ha="left",
        va="top",
        arrowprops=dict(arrowstyle="->", lw=1.6),
        fontsize=11,
    )

    ax.set_xlim(0.5, T_max + 0.5)
    ax.set_xlabel("Round of agreement T")
    ax.set_ylabel("Density")
    ax.set_title(f"Distribution of time to agreement (conditional on agreement; {policy})")

    fig.tight_layout()
    path = os.path.join(outdir, fname)
    fig.savefig(path, dpi=220)
    plt.close(fig)
    return path

def fig4_combo(df_cellF, mix_equal, mix_Hheavy, outdir,
              beta_line=3.0, betas=(1.0, 3.0, 6.0), G=200, eps=0.01,
              fname="fig4_combo_evolution_and_dominance.png"):

    def _policy_fitness_from_cellF(df_cellF, mix):
        piv = df_cellF.pivot(index="task", columns="policy", values="F")
        tasks = list(piv.index)
        pols = list(piv.columns)
        w = np.array([mix.get(t, 0.0) for t in tasks], dtype=float)
        w = w / w.sum()
        Fpol = (w[:, None] * piv.values).sum(axis=0)
        return pols, Fpol.astype(float)

    def evolve_fermi(F, beta=3.0, G=200, eps=0.01):
        F = np.asarray(F, dtype=float)
        K = len(F)
        P = np.ones(K) / K
        traj = np.zeros((G + 1, K), dtype=float)
        traj[0] = P.copy()
        for g in range(G):
            meanF = float((P * F).sum())
            weights = P * np.exp(beta * (F - meanF))
            Pp = weights / weights.sum()
            P = (1.0 - eps) * Pp + eps * (np.ones(K) / K)
            P = P / P.sum()
            traj[g + 1] = P
        return traj

    mixes = [("equal", mix_equal), ("H-heavy", mix_Hheavy)]

    pols_L, F_L = _policy_fitness_from_cellF(df_cellF, mix_equal)
    traj = evolve_fermi(F_L, beta=beta_line, G=G, eps=eps)

    margins = np.zeros((len(mixes), len(betas)), dtype=float)
    top_labels = [["" for _ in betas] for __ in mixes]

    for i, (_mix_name, mix) in enumerate(mixes):
        pols_i, F_i = _policy_fitness_from_cellF(df_cellF, mix)
        for j, b in enumerate(betas):
            tr = evolve_fermi(F_i, beta=b, G=G, eps=eps)
            last = tr[-1]
            order = np.argsort(-last)
            top, runner = float(last[order[0]]), float(last[order[1]])
            margins[i, j] = top - runner
            top_labels[i][j] = pols_i[order[0]]

    fig = plt.figure(figsize=(13.6, 4.8))
    gs = fig.add_gridspec(1, 2, width_ratios=[1.45, 1.0], wspace=0.32)

    axL = fig.add_subplot(gs[0, 0])
    for k, name in enumerate(pols_L):
```

```
        axL.plot(traj[:, k], label=name, linewidth=2.2)
    axL.set_xlabel("Generation")
    axL.set_ylabel("Policy share")
    axL.set_title(f"Evolutionary selection (beta={beta_line:.1f}, equal mix; eps={eps:.2f})")
    axL.set_ylim(-0.02, 1.02)

    # ===== legend centered INSIDE axL (your request) =====
    leg = axL.legend(
        loc="center",
        bbox_to_anchor=(0.55, 0.55),
        frameon=True,
        ncol=1,
        fontsize=11,
        handlelength=2.4
    )
    leg.get_frame().set_alpha(0.90)
    leg.get_frame().set_facecolor("white")

    axR = fig.add_subplot(gs[0, 1])
    im = axR.imshow(margins, aspect="auto", origin="upper")
    axR.set_title("Dominance margin\n(top share - runner-up)")
    axR.set_yticks(np.arange(len(mixes)))
    axR.set_yticklabels([m[0] for m in mixes])
    axR.set_xticks(np.arange(len(betas)))
    axR.set_xticklabels([f"beta={b:g}" for b in betas])

    for i in range(len(mixes)):
        for j in range(len(betas)):
            axR.text(j, i, f"{margins[i, j]:.2f}\n({top_labels[i][j]})",
                     ha="center", va="center", fontsize=11)

    fig.colorbar(im, ax=axR, fraction=0.046, pad=0.04)

    fig.tight_layout()
    path = os.path.join(outdir, fname)
    fig.savefig(path, dpi=220)
    plt.close(fig)
    return path

# ========= main =========
def main():
    ap = argparse.ArgumentParser()
    ap.add_argument("--out", type=str, default="./out_review", help="output directory")
    ap.add_argument("--N", type=int, default=240, help="episodes per cell")
    ap.add_argument("--seed", type=int, default=123, help="base seed")
    ap.add_argument("--B", type=int, default=2000, help="bootstrap replicates")
    ap.add_argument("--eps_prog", type=float, default=0.0, help="progress threshold eps_Gamma")
    ap.add_argument("--evo_G", type=int, default=200, help="generations for Fig4")
    ap.add_argument("--evo_eps", type=float, default=0.01, help="epsilon mixing for Fig4")
    ap.add_argument("--fig3_policy", type=str, default="P0_neutral", help="policy for Fig3 aggregation")

    args, _unknown = ap.parse_known_args()

    os.makedirs(args.out, exist_ok=True)
    episodes_dir = os.path.join(args.out, "episodes")
    os.makedirs(episodes_dir, exist_ok=True)

    # paper weights (fixed)
    w = [1.0, 0.6, 0.2, 0.2]

    tasks = list(TASKS.keys())
    pols = list(POLICIES.keys())
    mix_equal = task_mix_equal(tasks)
    mix_H = task_mix_Hheavy(tasks)

    with open(os.path.join(args.out, "run_config.json"), "w", encoding="utf-8") as f:
        json.dump(dict(N=args.N, seed=args.seed, B=args.B, weights=w,
                       eps_prog=args.eps_prog, evo_G=args.evo_G, evo_eps=args.evo_eps,
```

```
                fig3_policy=args.fig3_policy),
            f, ensure_ascii=False, indent=2)

    rows = []
    for pol_name, pol in POLICIES.items():
        for task_name, envP in TASKS.items():
            P = Params(**{**asdict(envP), **pol, "eps_prog": args.eps_prog})
            cell_seed = _stable_int_seed(args.seed, task_name, pol_name)
            ep_df = run_cell_episode_df(P, N=args.N, seed=cell_seed)

            ep_path = os.path.join(episodes_dir, f"episodes_{pol_name}__{task_name}.csv")
            ep_df.to_csv(ep_path, index=False)

            met = metrics_from_episode_df(ep_df)
            F = score(met, w)
            rows.append(dict(policy=pol_name, task=task_name, F=F, **met))

    df = pd.DataFrame(rows)
    df.to_csv(os.path.join(args.out, "per_task_metrics.csv"), index=False)

    summary_equal = df.groupby("policy").agg(
        overall_F=("F", "mean"),
        Pr_mean=("Pr","mean"),
        ET_mean=("ET","mean"),
        Ineq_mean=("Ineq","mean"),
        Vol_mean=("Vol","mean"),
        Pr_std=("Pr","std"),
    ).reset_index()
    summary_equal["Pr_CV"] = summary_equal["Pr_std"]/(summary_equal["Pr_mean"]+1e-9)
    summary_equal["Robust"] = np.exp(-summary_equal["Pr_CV"])
    summary_equal = summary_equal.drop(columns=["Pr_std"]).sort_values("overall_F", ascending=False).reset_index(dr
    summary_equal.to_csv(os.path.join(args.out, "scoreboard_equal.csv"), index=False)

    Fpol_H = policy_overall_from_cellF(df[["policy","task","F"]], mix_H).rename("overall_F")
    summary_H = df.groupby("policy").agg(
        Pr_mean=("Pr","mean"),
        ET_mean=("ET","mean"),
        Ineq_mean=("Ineq","mean"),
        Vol_mean=("Vol","mean"),
        Pr_std=("Pr","std"),
    ).reset_index()
    summary_H = summary_H.merge(Fpol_H.reset_index().rename(columns={"index":"policy"}), on="policy", how="left")
    summary_H["Pr_CV"] = summary_H["Pr_std"]/(summary_H["Pr_mean"]+1e-9)
    summary_H["Robust"] = np.exp(-summary_H["Pr_CV"])
    summary_H = summary_H.drop(columns=["Pr_std"]).sort_values("overall_F", ascending=False).reset_index(drop=True)
    summary_H.to_csv(os.path.join(args.out, "scoreboard_Hheavy.csv"), index=False)

    boot_seed = _stable_int_seed(args.seed, "bootstrap")
    overallF_boot_equal, diff_boot_equal = bootstrap_overallF_from_files(
        episodes_dir=episodes_dir, pols=pols, tasks=tasks, w=w, mix=mix_equal,
        B=args.B, seed=boot_seed
    )
    overallF_boot_H, diff_boot_H = bootstrap_overallF_from_files(
        episodes_dir=episodes_dir, pols=pols, tasks=tasks, w=w, mix=mix_H,
        B=args.B, seed=boot_seed + 1
    )

    rows_ci_equal = []
    for j, pol in enumerate(pols):
        m = float(overallF_boot_equal[:, j].mean())
        lo, hi = ci_percentile(overallF_boot_equal[:, j])
        rows_ci_equal.append(dict(policy=pol, mean=m, ci_lo=lo, ci_hi=hi))
    pd.DataFrame(rows_ci_equal).sort_values("mean", ascending=False).to_csv(
        os.path.join(args.out, "bootstrap_overallF_equal.csv"), index=False
    )

    rows_ci_H = []
    for j, pol in enumerate(pols):
```

```
        m = float(overallF_boot_H[:, j].mean())
        lo, hi = ci_percentile(overallF_boot_H[:, j])
        rows_ci_H.append(dict(policy=pol, mean=m, ci_lo=lo, ci_hi=hi))
    pd.DataFrame(rows_ci_H).sort_values("mean", ascending=False).to_csv(
        os.path.join(args.out, "bootstrap_overallF_Hheavy.csv"), index=False
    )

    if diff_boot_equal is not None:
        m = float(diff_boot_equal.mean()); lo, hi = ci_percentile(diff_boot_equal)
        pd.DataFrame([dict(diff="P0_neutral - P2_weak", mean=m, ci_lo=lo, ci_hi=hi)]).to_csv(
            os.path.join(args.out, "bootstrap_diff_P0minusP2_equal.csv"), index=False
        )
    if diff_boot_H is not None:
        m = float(diff_boot_H.mean()); lo, hi = ci_percentile(diff_boot_H)
        pd.DataFrame([dict(diff="P0_neutral - P2_weak", mean=m, ci_lo=lo, ci_hi=hi)]).to_csv(
            os.path.join(args.out, "bootstrap_diff_P0minusP2_Hheavy.csv"), index=False
        )

    df_cellF = df[["policy","task","F"]].copy()
    pA = figA_overall_bar(df_cellF, mix=mix_equal, outdir=args.out, fname="figA_overall_score.png")
    pB = figB_task_policy_heatmap(df_cellF, outdir=args.out, fname="figB_per_task_heatmap.png")
    p3 = fig3_T_histogram_aggregate(
        episodes_dir=episodes_dir, policy=args.fig3_policy, tasks=tasks,
        T_max=BASE.T_max, outdir=args.out, fname="fig3_T_distribution.png"
    )
    p4 = fig4_combo(
        df_cellF=df_cellF, mix_equal=mix_equal, mix_Hheavy=mix_H, outdir=args.out,
        beta_line=3.0, betas=(1.0, 3.0, 6.0), G=args.evo_G, eps=args.evo_eps,
        fname="fig4_combo_evolution_and_dominance.png"
    )

    print("== DONE ==")
    print("dir:", os.path.abspath(args.out))
    print("episodes_dir:", os.path.abspath(episodes_dir))
    print("per_task_metrics.csv:", os.path.join(args.out, "per_task_metrics.csv"))
    print("scoreboard_equal.csv:", os.path.join(args.out, "scoreboard_equal.csv"))
    print("scoreboard_Hheavy.csv:", os.path.join(args.out, "scoreboard_Hheavy.csv"))
    print("bootstrap_overallF_equal.csv:", os.path.join(args.out, "bootstrap_overallF_equal.csv"))
    print("bootstrap_overallF_Hheavy.csv:", os.path.join(args.out, "bootstrap_overallF_Hheavy.csv"))
    print("bootstrap_diff_P0minusP2_equal.csv:", os.path.join(args.out, "bootstrap_diff_P0minusP2_equal.csv"))
    print("bootstrap_diff_P0minusP2_Hheavy.csv:", os.path.join(args.out, "bootstrap_diff_P0minusP2_Hheavy.csv"))
    print("FigA:", pA)
    print("FigB:", pB)
    print("Fig3:", p3)
    print("Fig4:", p4)

if __name__ == "__main__":
    main()
 }
```
